# Spatial and Temporal Analysis, and Machine Learning-Based Prediction of PCB Water Concentrations in U.S. Natural Water Systems

**DOI:** 10.1021/acsestwater.4c00542

**Published:** 2024-12-17

**Authors:** Andres Martinez, Keri C. Hornbuckle, Michael P. Jones, Brian D. Westra

**Affiliations:** † Department of Civil & Environmental Engineering, IIHR-Hydroscience and Engineering, The University of Iowa, Iowa City, Iowa 52242, United States; ‡ Department of Biostatistics, The University of Iowa, Iowa City, Iowa 52242, United States; § University of Iowa Libraries, The University of Iowa, Iowa City, Iowa 52242, United States

**Keywords:** PCB, water concentration, prediction, machine learning, half-lives, water systems, U.S., data wrangling.

## Abstract

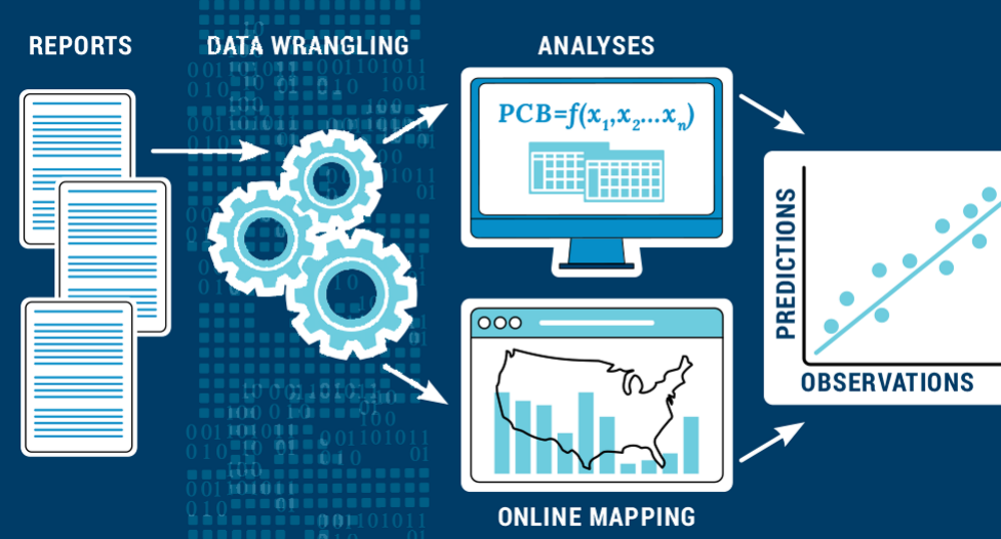

Data on dissolved phase water concentrations of polychlorinated biphenyls (PCBs) from 32 locations across the U.S. were compiled from reports, Web sites, and peer-reviewed papers, spanning 1979–2020, resulting in 5132 individual samples. Data wrangling enabled the organization and analysis of this extensive data set. Most samples originated from PCB Superfund sites like the Fox, Hudson, and Kalamazoo rivers, New Bedford Harbor, and Lake Michigan. ΣPCB concentrations ranged from 10°–10^7.3^ pg/L, while individual congener medians ranged from nondetected to 380 pg/L. Non-Aroclor congeners, e.g., PCBs 11, 67, and 68, were also reported. Using a machine learning technique, a Random Forest model accurately predicted the temporal and spatial occurrence of dissolved PCBs, achieving Pearson correlations greater than 0.87 for the Anacostia, Fox, Hudson, Kalamazoo, Passaic, and Spokane rivers, Chesapeake Bay, and New Bedford Harbor. These models can be used to forecast PCB concentrations. Through a linear mixed-effects model, half-lives of approximately 8 years for ΣPCB and individual congeners were determined, but the resulting half-lives showed considerable variability. An interactive map of ΣPCB was created. This investigation highlights the need for additional sampling in PCB-contaminated sites that may expose communities to airborne PCBs, and in other locations, to enhance our understanding of PCB occurrence and distribution.

## Abstract 2

Compilation of U.S. PCB water samples (1979−2020) reveals occurrence and trends; a Random Forest model accurately predicts water PCB concentrations.

## Introduction

Since the implementation of more stringent environmental regulations for persistent organic pollutants (POPs) around the world, which resulted in production bans, source reductions, and controls, anthropogenically impacted sediments have shifted from sinks to sources of POPs.^1^ This shift has had a direct impact on the quality of waters above them. Polychlorinated biphenyls (PCBs), one of the initial dirty dozen POPs classified in the Stockholm Convention,^2^ are commonly found in both fresh and saltwater sediments. In the U.S. alone, they are associated with more than 50 Superfund sites and possibly more unclassified contaminated sites.^3^ The U.S. Environmental Protection Agency (USEPA) has primarily focused on sediment PCB levels and how they impact biota and potential human consumption of contaminated fish and seafood.^4^

To date, the potential for airborne PCB emissions from Superfund sites has not been fully explored. This is particularly concerning for communities living near these sites as they may be exposed to PCBs through inhalation, in addition to potential exposure through fish and seafood consumption. Predicting community exposure to airborne PCBs released from contaminated water bodies should be a factor in prioritizing sites and remediation efforts. The Iowa Superfund Research Program (https://iowasuperfund.uiowa.edu/) has focused on this important issue, addressing concerns in communities such as New Bedford Harbor, MA, East Chicago, IN, and Portland Harbor, OR. Our research has demonstrated that PCBs present in water bodies can be released into the air, highlighting the need to assess and mitigate inhalation risks to protect public health.^5−8^

Dissolved water concentrations of PCBs are crucial for making emission estimations, and the USEPA typically monitors them at designated Superfund sites through contractors. Unfortunately, significant amounts of these data are not publicly available or easy to access. Similar challenges exist with scientific peer-reviewed papers, where data are often not tabulated, even in Supporting Information or online repositories. Moreover, the lack of standardized data and metadata formats and structures further complicates data usability and interoperability.

Our primary objective was to compile a comprehensive U.S. dissolved PCB water concentration data set and make it publicity accessible, aligned with the FAIR principles (Findable, Accessible, Interoperable, and Reusable), as outlined by Rocca-Serra et al.^9^ Although machine-readable metadata is a key element of FAIR,^10^ our focus was on compiling data from diverse sources into a more accessible structure. By creating this data set, we aimed to identify available surface water PCB concentrations, assess the feasibility of integrating diverse data sources, develop statistical models for future predictions, evaluate model accuracy, identify potential temporal trends, and create a publicly accessible interactive web-based map. The interactive map displays surface water PCB concentrations and other relevant information, allowing users to explore the data and their temporal and spatial distribution.

## Materials and Methods

### Data Collection

The obtained data on PCB water concentrations were not aligned with the FAIR principles. The data were primarily obtained from USEPA reports and their contractors (from 1979 to 2020) through Freedom of Information Act (www.foiaonline.gov) requests. These requests were focused on regions with known PCB Superfund Sites, as well as the 10 USEPA Regions, state environmental agencies, such as the Departments of Natural Resources (DNRs), and Departments of Environmental Quality (DEQs).

Data acquisition often proved to be time-consuming, marked by inconsistent availability and accessibility from sources. Most of the data were obtained as PDF-formatted tables in reports. In a few cases, data were available as comma-separated values (CSV) files via Web sites such as ph-public-data.com and the Great Lakes Environmental Database Query System (GLENDA) for the Lake Michigan Mass Balance project. These included data from tributaries to Lake Michigan and from the National Water Quality Monitoring Council. Other data were provided on CDs (Excel and Access files), such as the Hudson River data set.

In the absence of tabular data reported in peer-reviewed papers, some data were presented as plots, making it labor-intensive to extract specific values and increasing uncertainty. We found only a few peer-reviewed papers with data reported in easily extractable tables. In most cases, only ΣPCB levels were provided.^11−17^ To our knowledge, our research group is the only one to upload data to an online repository.^18^

Inconsistent reporting formats and machine-unfriendly data structures, such as varying file formats and column headings, as well as the lack of clear and easily accessible metadata (e.g., data dictionaries), hinder the interoperability and reusability of the data. Additionally, variations in analytical methods, particularly in the GC columns employed, result in discrepancies in the quantities of individual and coeluting congeners. This variability complicates the establishment of a standardized list of PCB congeners for future analyses.

### Data Wrangling

Data provided as tables in PDF files required extensive work to convert into tabular data files like CSVs. We used a PDF converter when possible; however, in some cases, manual transcription of the data was necessary. In situations wherein the data originated from CSV or Access files, we created custom R codes to read, organize, and generate new CSV files. To address cases where data sets provided different congener lists, we developed a unique congener list comprising 104 individual and coeluting PCB congeners (Table S1). When congeners were reported individually, we summed them to create these coeluting groups. This task was accomplished using a combination of R and manual data entry methods. For samples that only reported total Aroclor data, the code extracted those Aroclor values, indicating which Aroclor mixture was reported.

To achieve a consistent approach to location information across all data, we defined the location as a geographical area encompassing multiple sampling sites and focused on the dissolved phase of water, as defined by water passed through a filter. It is probable that different filter sizes were used in collecting water samples. Most samples were collected using a judgmental approach,^19^ targeting high PCB contamination sites, so to date, there has not been a nationwide effort to determine PCB contamination in surface waters.

In the compiled data file, each row represents a unique sample, and the columns represent the sampling information (metadata) and individual congener values. Sampling information includes the source of the data, the EPA Region, the state or province, the site name, sampling date, latitude, longitude, units (pg/L), the measured phase, the EPA analytical method, and whether the analytical method was Aroclor or congener-specific. The compiled data also include seven columns for the values for the most common Aroclor mixtures (i.e., 1016, 1221, 1232, 1242, 1248, 1254, and 1260), to indicate which Aroclor was measured, along with the total concentration. We created a unique site identifier, ‘SiteID,’ which is comprised of the first five characters ‘WCPCB,’ followed by a three-character abbreviation of the location name, and then a three-digit site number. For instance, ’WCPCB-FOX003’ represents the Fox River location and site number 3. We created a unique sample identifier, ‘SampleID,’ by combining the SiteID with the sampling date. If duplicate samples were collected at the same site and day, we added a replicate number to the end of the SampleID. For example, ‘WCPCB-FOX003–20050803.1’ represents the Fox River site 3, sampled on August 3, 2005, with a single replicate (Figure S1). Initially, we compiled 45,980 individual samples. However, due to replicate analytical measurements conducted using EPA Aroclor methods, where between six and seven Aroclor mixtures were reported regardless of their presence in the sample, we retained only the highest value representing the Aroclor mixture known to have contaminated the location. Additionally, samples reported as below or equal to the detection limit for Aroclors or total PCBs were excluded, as detailed information about the detection limit or its determination was often unavailable or unclear.

Additionally, to minimize uncertainty in individual PCB values, we opted against estimating congener-specific data from the Aroclor measurements. This screening resulted in 5132 individual samples being retained for analysis, generating a matrix of 5132 rows by 126 columns, resulting in 646,632 metadata/values, from which 574,784 data points are PCB concentration data. The final data set is published open access in the Pangaea repository.^20^ Furthermore, all the R codes created here to analyze, model, and generate the plots and maps are freely available in the Zenodo repository.^21^

### Data Visualization and Statistical Analysis

We used R (version 4.2.1) to analyze the data and generate graphs and maps. Individual, total PCB (sum of 104 individual and coeluting PCB congeners or ΣPCB), and total Aroclor concentrations were mostly consistent with a log-normal distribution; therefore, they were log_10_-transformed for further analysis.

A machine learning model, Random Forest, was used to develop a prediction model for total, individual, and coeluting groups of PCB congeners. The model will predict total, individual, and coeluting groups of PCB congeners' water concentrations at specific locations and times. We utilized two R packages: ranger and caret, which offer flexibility to select various parameters, including the number of trees, the number of variables randomly sampled as candidates at each split, the split rule for decision trees, and the minimum size of terminal nodes in the trees for optimization purposes. To develop the model, 80% of the collected data were used for training and the remaining 20% for testing. We first selected individual and coeluting groups of PCB congeners that had a detection frequency of 70% or greater. For those selected PCBs, we used all available measurements and not just the ones that exceeded the 70% detection threshold. PCBs that did not meet the 70% detection frequency were excluded from the analysis.

As input covariates to the model, we included time, expressed in Julian days; season, represented by four categories (i.e., summer, winter, spring, and autumn); water flow; and water temperature, if available. Whenever possible, daily averages of water flow and water temperature for the sample locations were collected from USGS stations by using the dataRetrieval R package. Water temperatures for the Great Lakes and Chesapeake Bay were obtained from the National Data Buoy Center. Euclidean distance from the sample site to known source(s) was included as a spatial covariate. In locations where no source(s) were identified, the most northern, western, or eastern sample was selected as a reference point, depending on the water flow direction, and the distance was estimated. For locations with a more circular sampling approach (e.g., Lake Michigan, Chesapeake Bay), a centroid point was estimated, and the distance to the sampling sites was calculated.

The predicted model performance was assessed using the test data and the following statistical parameters: root-mean-square error (RMSE), the Pearson correlation between predicted and observed values, and an error defined as the ratio between the predicted and observed values on the original scale, with a threshold factor of 2.

Preliminary results using the entire data set for ΣPCB yielded an RMSE of 0.40, a Pearson correlation of 0.93, and a factor of 2, 74% of the time. The two parameters that significantly improved the model were the number of trees and the minimum size of terminal nodes in the trees. However, beyond a certain threshold (number of trees = 5000, number of variables randomly sampled as candidates at each split = 3, split rule for decision trees = variance reduction, and minimum size of terminal nodes in the trees = 5), the incremental improvements became minimal. Considering individual PCB congeners, where a 70% or more detection frequency yielded 61 individual congeners, the Random Forest model yielded an average RMSE of 0.35 (±0.05), an average Pearson correlation of 0.92 (±0.02), and predicted and observed values within a factor of 2, 76 (±5) % of the time. Figure S2 depicts predicted vs observed ΣPCB and individual PCB values. In both cases, only time, site, and seasons were included as covariates. Because it was not possible to include water flow, water temperature, and distance as covariates, we decided to carry out predictive modeling for each location that had at least 35 samples. Through this approach, which is location-specific, the rest of the covariates were included, thus significantly improving the robustness of the model. Furthermore, we were able to reduce the large variability in sampling and analytical methods performed by various contractors for the different locations.

Additionally, we focused on temporal trends to estimate half-lives (*t*_0.5_) by assessing concentrations using linear mixed-effects (LME) models. The same covariates were used, except for the distance to a source/site. As previously mentioned regarding the Random Forest approach, initial findings from these parametric models, when aggregating all samples, highlight the importance of conducting models for each location and incorporating the sampling sites’ effect as a random factor. For example, the explained total variability increased from 0.03 to 0.88 when accounting for this random site effect in ΣPCB (Figure S3). Therefore, the LME model was selected, using the following model structure (eq 1):^22^
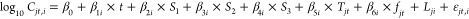
1where *jt* indexes the sample taken at site *j* at time *t*, and *i* indexes the specific PCB congener or ΣPCB. *C*_*jt,i*_ is the water concentration of the *i*th PCB or ΣPCB in pg/L, the β_*i*_’s are the regression coefficients for modeling the *i*th PCB or ΣPCB, t is the time in days when the sample was collected, *S*_1_, *S*_2_, and *S*_3_ are the seasons in relation to the fourth season, *T*_*jt*_ is the water temperature in Kelvin, *f*_*jt*_ is the water flow in m^3^/s, *L*_*ji*_ is the random effect for site *j*, and ε_*jt,i*_ is the residual error term for the *i*th PCB or ΣPCB. Furthermore, we wanted to explore the idea of describing the flow effect as a quadratic function, as noted by Marti and Armstrong.^23^ Here, β_6*i*_ × *f*_*jt*_ was replaced by β_60*i*_ + β_61*i*_ × *f*_*jt*_ + β_62*i*_ × *f*_*jt*_^2^. Despite the LME yielding a high *R*^2^ (0.88), two assumptions were not satisfied. First, the residuals from the model did not show a normal distribution, as confirmed by a Q–Q plot, and second, the residuals did not have constant variance (Figure S3). Similar results were yielded for individual PCB congeners. As indicated for the Random Forest Model, water flow and temperature were not included in this preliminary approach. Thus, we decided to perform this analysis per location, such as the Hudson River, the Fox River, and so forth, where most of the time, water flow and temperature were available for the sampling period. Here, both assumptions were satisfied for ΣPCB and a selected group of individual congeners at specific locations (Table S2). Based on separate LME models (eq 1) for the locations, location-specific half-lives for ΣPCB and the i-th PCB were calculated as follows:^24^
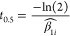
2and the error
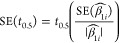
3where SE(*β̂*_1*i*_) is the standard error of *β̂*_1*i*_. We only reported *t*_0.5_ for ΣPCB and individual congeners when β_1*i*_ from eq 1 was statistically significant (*p*-value <0.05). If samples were known to be collected during dredging operations or from background sites, they were not included, as they were considered as outliers.

## Results and Discussion

### General Overview

Data for surface water samples were available from 32 locations, covering 22 states, the District of Columbia, and Ontario, Canada, spanning the years 1979 to 2020. Most of the samples were from rivers and creeks (freshwater), with a few estuaries/bays, such as Chesapeake Bay and New Bedford Harbor. Samples from lakes included those from the Great Lakes region—especially Lake Michigan, Onondaga Lake (NY), and Lake Washington (WA). The number of samples per location ranged from 1 to 1395, with a median of 34. Notably, rivers such as the Housatonic, Passaic, Kalamazoo, and Hudson, along with Lake Michigan, had over 400 samples each. The data set includes 795 sampling sites, with replicate sampling varying from 1 to 8. The top sampling site, with eight replicates, was North Farmer St. along the Kalamazoo River in Michigan.

**Figure 1:**
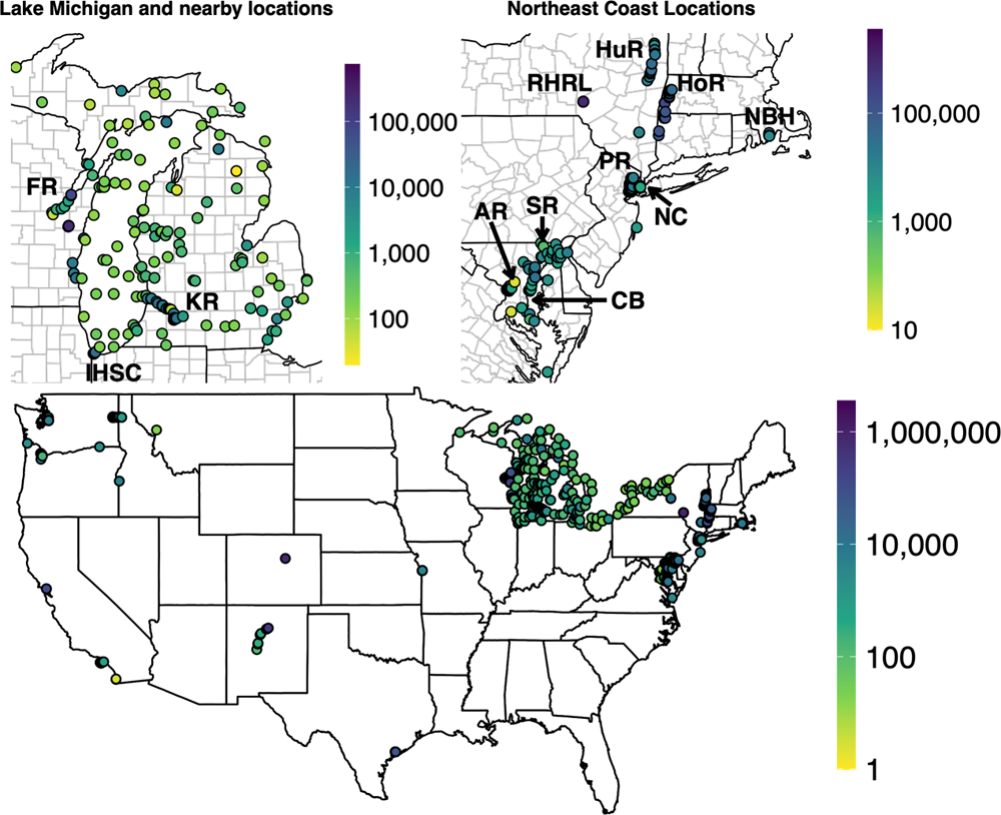
Spatial distribution of mean concentration of ΣPCB (pg/L) per site (see Figure S1 for SiteID examples). Aroclor and individual congeners data were used (*n* = 5132). Top left map shows Lake Michigan and nearby locations, where FR = Fox River, KR = Kalamazoo River, and IHSC = Indiana Harbor and Ship Canal. Top right map shows Northeast locations, where HuR = Hudson River, HoR = Housatonic River, RHRL = Richardson Hill Road Landfill, NBH = New Bedford Harbor, PR = Passaic River, NC = Newtown Creek, SR = Susquehanna River, AR = Anacostia River, and CB = Chesapeake Bay.

Sample locations for the U.S. represented in the data are not evenly distributed and tend to cluster in two main areas: around Lake Michigan and a smaller cluster in the Northeast Coast, encompassing Delaware, Maryland, Massachusetts, and New York (Figures 1 and S4). This spatial pattern aligns with the presence of established PCB Superfund sites, including New Bedford Harbor, the Hudson River, the Passaic River, the Housatonic River, Green Bay/Lake Michigan, and the Kalamazoo River. The Lake Michigan Mass Balance project was also a source of data from extensive sampling due to the heavy usage of Aroclors by facilities located near this water body. Notably, this spatial distribution of the sampling locations is not associated with Aroclor manufacturing or proximity to densely populated areas. In the context of Aroclor manufacturing, only particulate water samples were found in Anniston, Alabama. This absence of dissolved phase reporting may also explain why other iconic PCB contamination sites are missing from our data set. As mentioned previously, we were primarily interested in the dissolved phase rather than in whole or particulate water phases.

We observed a distinct temporal shift in the chemical methods used for analyzing water samples, a transition from Aroclor to congener-specific methods. From 1979 to 2001, the Aroclor method predominantly quantified most samples, except during the Lake Michigan Mass Balance project, which was developed between 1993 to 1995. Between 2001 and 2010, both methods were employed almost equally. Starting in 2011, the congener-specific method became the predominant method for sample analysis. However, despite this trend, two samples from the Housatonic River in 2020 were analyzed using the Aroclor method (Figure S5). The congener-specific method does not necessarily cover all 209 individual congeners. For instance, samples from New Bedford Harbor were analyzed using an NOAA PCB congener method, resulting in the identification of only 18 individual congeners. The individual PCB congener EPA Method 1668 was introduced in 1999,^25^ while some laboratories continued to use EPA Aroclor methods (608 and 8082). The continued use of the Aroclor methods may stem from the high costs associated with employing an isotope dilution and internal standard method with HRGC/HRMS, which may not have been mandated by local environmental agencies.

Slightly less than half of the data we compiled were for Aroclor mixtures (2022/5132 = 39%), with the rest as individual PCB congeners. Among the samples reported as Aroclor mixtures, Aroclors 1242, 1254, and 1260 were the most frequently represented in the data, appearing 17, 23, and 48% of the time, respectively, while Aroclor 1221 was the least reported at 0.8%. Aroclor reporting was largely driven by the number of samples from the Housatonic River, which were predominantly contaminated with Aroclors 1254 and 1260, and samples from the Kalamazoo River containing Aroclor 1242. Of the samples that reported individual PCB congeners (3110/5132 = 61%), all 104 individual or coeluting congeners were detected at least 3% of the time. Notably, PCBs 78, 80, 186, and 192 were the congeners detected at the minimum frequency of 3%, while coeluting congener PCBs 20 + 21 + 28 + 31 + 33 + 50 + 53 yielded a maximum of 86% of the time. Interestingly, non-Aroclor individual congeners, as defined by Koh et al.,^26^ were also detected. These included PCBs 11 (28%), 14 (5%), 23 (5%), 35 (29%), 38 (10%), 39 (24%), 54 (27%), 57 (22%), 58 (20%), 67 (37%), 68 (25%), 78 (3%), 79 (25%), 94 (21%), 103 (26%), 104 (13%), 120 (13%), 121 (4%), 127 (5%), 148 (8%), 175 (18%), 181 (7%), 184 (7%), 186 (3%), 188 (6%), 189 (19%), 191 (20%), 192 (3%), and 204 (4%). It is surprising to find a relatively high detection frequency for PCB 67 (37%) among all these non-Aroclor congeners, particularly considering the unknown potential sources of PCB 67 and the fact that its percentage in commercial Aroclor mixtures is less than 0.18%.^27^ The median detection frequency for all 104 congeners and coeluting congeners was 41%. Figure S6 displays the detection frequencies for all individual PCB congeners.

### Total and Individual PCB Concentrations

**Figure 2:**
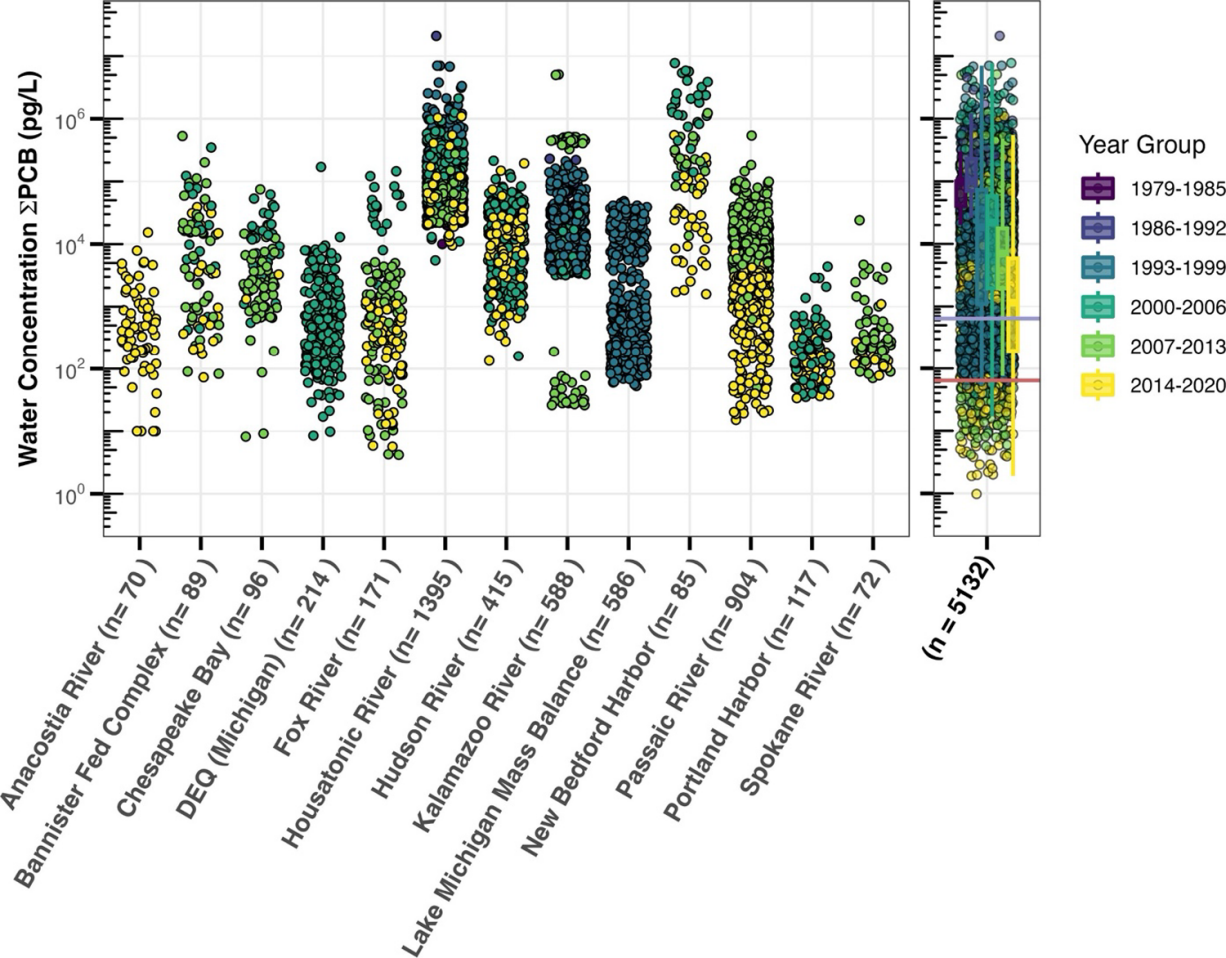
ΣPCB water concentration for selected sites (left plot) and all samples (right plot) in pg/L. The year of sampling is represented by color in intervals of 6 years. Left plot shows 94% of the total collected data. Housatonic and Kalamazoo rivers were analyzed using an Aroclor method, while the other locations used congener-specific methods. The purple line in the right panel represents the 640 pg/L Water Quality Criterion for Human Health from fish consumption associated with an incremental cancer risk of 10^–5^, and the red line represents the Water Quality Criterion of 64 pg/L associated with an incremental cancer risk of 10^–6^.^28^

ΣPCB concentrations in individual samples ranged over 7 orders of magnitude with a median of 10,400 pg/L and an interquartile of 47,000 pg/L (Figure 2). Although very high concentrations (10^6.7^–10^7.3^ ppm/L) were detected, they were infrequent. The top 10 highest concentration samples, exceeding 10^6.7^ pg/L, were collected in the Housatonic and Kalamazoo rivers, as well as New Bedford Harbor (Figure 2). The highest recorded sample was from the Housatonic River, collected in March 1991 at the St. Bridge site. Of the total samples collected, 79% exceeded the Water Quality Criterion for Human Health associated with an incremental cancer risk of 10^–5^, set at 640 pg/L for ΣPCB from fish consumption. Ninety-six percent of the samples exceeded the Water Quality Criterion associated with an incremental cancer risk of 10^–5^, set at 64 pg/L.^28^ It is worth noting that most samples were collected from known PCB-contaminated sites, likely before any remediation process had occurred.

Examining selected locations, as illustrated in Figure 2, reveals the variability in ΣPCB levels between these locations. Additionally, within the same location, there is variability. For example, the Housatonic River and New Bedford Harbor concentrations are slightly higher than the rest, but their variability (∼3.5 log_10_ units) is smaller than that of the Fox, Kalamazoo, and Passaic rivers (∼4.5 log_10_ units), though larger than in Portland Harbor and the Spokane River (∼2 log_10_ units). Besides differences in PCB sources, sediment geochemistry, environmental and hydrological conditions impacting these locations, remediation activities can directly influence the variability between sites and within the same location. For instance, the highest values reported for the Kalamazoo River were collected during sediment dredging, while Portland Harbor and the Spokane River have not undergone dredging.

The individual PCB congener median concentrations of PCB congeners ranged from nondetected to 380 pg/L, with an average of the medians of 32 pg/L for all congeners. A few individual values were particularly high, such as PCBs 5 + 8 with a concentration of approximately 2,500,000 pg/L in a sample collected from New Bedford Harbor at Graham St. in September 2006. It is possible that these extremely high readings also captured particles after filtration. Figure S7 displays boxplots of individual PCB congener concentrations. A slight trend of decreasing medians is observed for congeners with an increasing number of chlorine substitutions. PCBs with more chlorine atoms tend to partition less into the water phase. However, the individual PCB concentrations exhibit considerable variation, with individual PCB values ranging with more than 4 log_10_ units, emphasizing the complexity of their occurrence in water systems.

When inspecting individual PCB congeners across selected locations, we observe a consistent pattern of variability both between different locations and within the same location. Notably, Aroclor (Figure S8) and non-Aroclor (Figure S9) congeners exhibit distinct patterns, suggesting different sources. For instance, Aroclor coeluting congeners, such as PCBs 5 + 8 and 20 + 21 + 28 + 31 + 33 + 50 + 53 (Figure S8), show a pattern of variability like that of ΣPCB. In contrast, non-Aroclor congeners such as PCBs 11 and 68 at the Hudson River exhibit levels similar to those observed at Portland Harbor during the 2014–2020 sampling period, unlike the much higher levels of ΣPCB measured in the Hudson River. Similarly, while Aroclor congeners have comparable values between Portland Harbor and the Spokane River, some non-Aroclor congeners, such as PCBs 35, 67, and 209 exhibit slightly higher concentrations in the Spokane River, except for PCB 68 (Figure S9).

While no uniform spatial distribution is evident for ΣPCB across the U.S., a clear hotspot is observed in the Northeast Coast. This hotspot is primarily associated with samples collected from the Housatonic River and New Bedford Harbor (dark blue dots in Figure 1). However, upon closer examination of specific locations, it is possible to observe some spatial distributions that may be explained by several phenomena (Figure S10). For instance, a clear North-to-South gradient is evident in samples taken from New Bedford Harbor. This was expected due to the location of the Aerovox facility, formerly situated in the Upper Harbor, known to be the primary source of Aroclor 1016 and 1242 in New Bedford Harbor.^29^ Concerning the Hudson River, elevated PCB levels are evident in the Upper Hudson River, where General Electric facilities in Hudson Falls and Fort Edward were situated, and Aroclors were extensively used.^30^ However, a hotspot appears to emerge to the south of these sites, possibly as a result of PCB transport in water and sediment flowing from both facilities, which are located to the north of this southern site, combined with increased sedimentation and flooding at that location.^31^ The Fox River data also exhibit a spatial distribution, with a hotspot located at the mouth of the Fox River and Lake Michigan. One possible explanation for this is that most of the collected samples were taken after sediment dredging, and the majority of PCB-contaminated sediment has been transported in the direction of Lake Michigan.^32^ In the Kalamazoo River, there is clearly a hotspot, which is correlated to a sediment dredging operation at Plainwell Dam during 2009–2010.

Individual PCB congeners exhibit a somewhat similar spatial distribution to ΣPCB, although fewer locations were available due to some reporting only Aroclor measurements (Figure S11). Most Aroclor congeners align with the spatial distribution of ΣPCB, but a few non-Aroclor congeners, including PCBs 11 and 67, exhibit distinct spatial signatures. Interestingly, high values for these non-Aroclor congeners were reported at the Bannister Federal Complex in Missouri. What adds further intrigue is that PCB 11 appeared in only samples from 2007 to 2019, with no reports in samples collected in 2004 and 2006. PCB 67 was reported in only one sample but with a relatively high value, approximately 400 pg/L. The source of these non-Aroclor congeners is unclear, but besides transformer PCB oils, caulking compounds and elastic sealants were also sources of PCB contamination at this site and could contain PCBs 11 and 67.^33^

### Random Forest Model Predictions for PCB Water Concentrations

**Figure 3:**
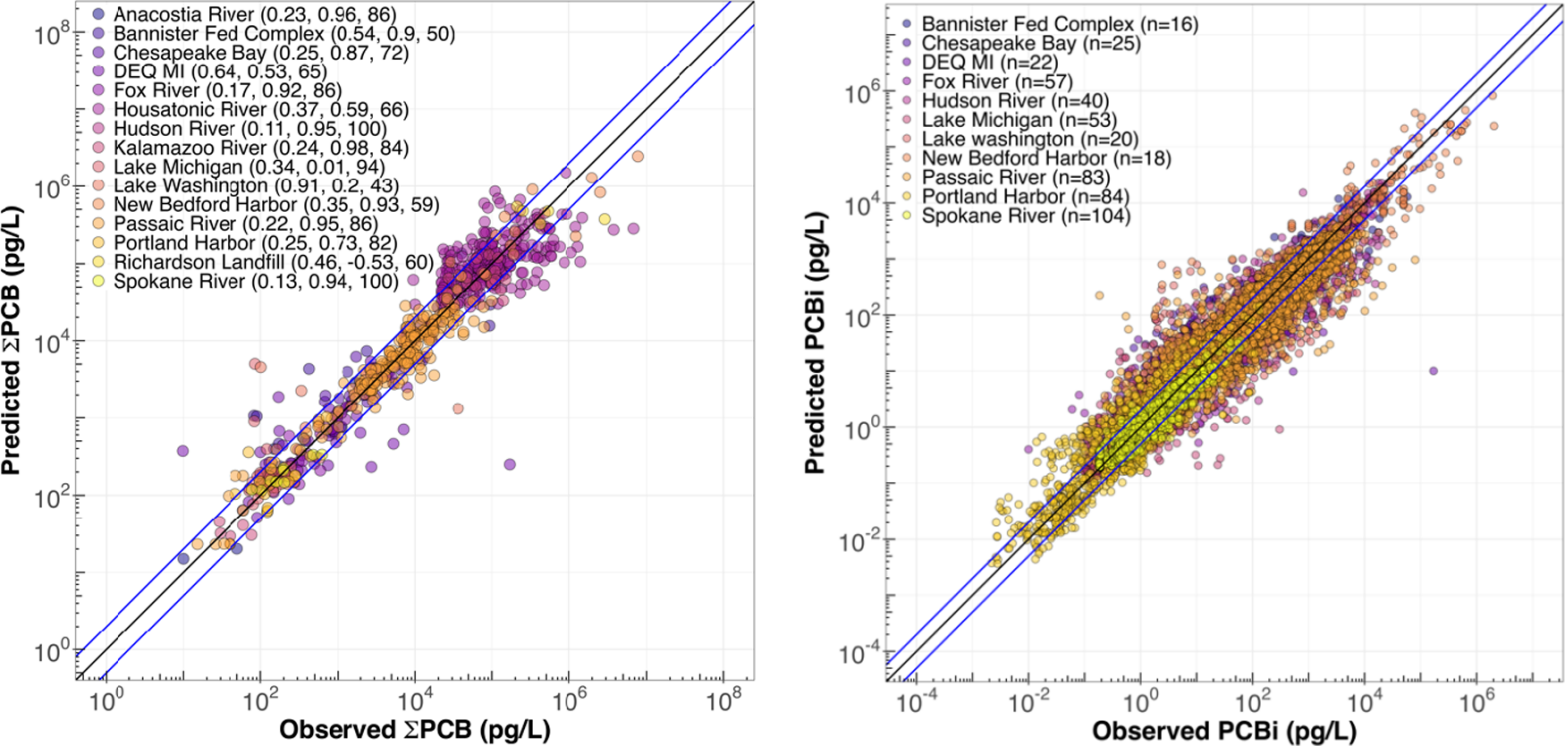
Random Forest predictions vs observations of ΣPCB (left plot) and individual PCB congeners (right plot) in pg/L. Numbers in parentheses on the left plot indicate RMSE, Pearson correlation, and a factor of 2 (%). Numbers in parentheses on the right plot indicate the number of predicted individual congeners per location. The black line represents the 1:1 line, and the blue lines represent the 1:2 and 2:1 lines (i.e., factor of 2). The right plot shows fewer locations due to some locations being only analyzed for Aroclor.

Focusing on individual locations and using covariates such as time, seasons, water flow, and water temperature when available, and distance to known sources or a reference site, the performance of the model for the concentration of ΣPCB varied. The RMSE ranged from 0.11 to 0.91, the Pearson correlation from −0.53 to 0.98, and the predictions were within a factor of 2 from the observations 43 to 100% of the time. Overall, the prediction model demonstrated better performance for river systems. For instance, the Anacostia, Fox, Hudson, Kalamazoo, Passaic, and Spokane rivers consistently yielded RSME values below 0.24, Pearson correlations above 0.92, and a ratio of factor of 2 more than 84% of the time. However, the Housatonic River did not perform as well (RSME = 0.37, Pearson = 0.60, and Factor of 2 = 66%). On the other hand, Chesapeake Bay and, especially, New Bedford Harbor showed good performance, with RMSE values below 0.35, Pearson correlations above 0.87, and a factor of 2 occurring 59% of the time. In the case of a more circular sampling approach, as observed in locations like Michigan and Washington lakes, as well as Richardson Hill Road landfill, the model exhibited poor performance, featuring a high RMSE (>0.33) and low Pearson correlations (<0.20). The latter outcomes could be due to including the distance to the center assumption as a covariate to the model and the low number of samples. Figure 3 displays the Random Forest predictions vs observations of ΣPCB in pg/L for selected locations (left plot). It is possible to observe that the model generally performs well; most samples fall within the band between the 1:2 and 2:1 lines, closely following the 1:1 line. There are a few exceptions; however overall, the model does not exhibit bias toward under or overpredictions.

For individual PCB congeners, performance remained consistent and comparable to that for ΣPCB, but it varied across different locations. For instance, the Hudson River displayed an RMSE ranging from 0.2 to 0.42, with a strong average Pearson correlation of 0.94, though individual correlations varied from 0.61 to 0.94. In contrast, Portland Harbor showed greater variability with an RMSE ranging from 0.2 to 0.86 and a Pearson correlation ranging from −0.64 to 0.9, with 50% of the samples above 0.5, reflecting less reliable predictions. Overall, RMSE values across all sites ranged from 0.09 to 1.0, with Pearson correlations from −0.64 to 0.99, with 90% of the samples above 0.5, and the factor of 2 accuracy varied significantly from 8 to 100%. Notably, most sites demonstrated effective predictions with average Pearson correlations of over 0.71, except for Lake Washington and Portland Harbor, which showed average correlations of −0.63 and −0.34, respectively. Figure 3, right plot, depicts the Random Forest predictions versus observations of individual PCB congeners in pg/L for selected locations.

### Temporal Analysis

There is no evident temporal trend of ΣPCB in the data set as a whole, except for a period of constant and narrow range values (∼2 log_10_ units) observed between 1979 and 1992 (Figure S12). However, if the data are grouped every 6 years, a decrease is observed but not significant (Figure 2). Data from 1997 to 2020 indicate a generally constant trend with a slight decrease but with a wide range in concentrations spanning approximately 5 to 6 log_10_ units. This constant trend is likely influenced by heavily sampled sites, including the Housatonic, Hudson, Kalamazoo, and Passaic rivers. However, a clear difference between different period samples collected is shown for the Passaic River, where, in general, all the high values were collected in early years (Figure 2). As mentioned earlier, examining each location individually reveals interesting temporal trends, not only for ΣPCB but also for individual congeners. For instance, the Fox River, New Bedford Harbor, and Portland Harbor exhibit a slight decrease in ΣPCB concentrations over time, as illustrated in Figure S10. In contrast, at some sites, such as the Hudson River and Chesapeake Bay, this trend is less clear and sometimes remains constant.

Concerning individual or coeluting PCB congeners, Aroclor congeners follow a very similar temporal trend from 1994 as ΣPCB, as shown for selected PCBs 5 + 8, 20 + 21 + 28 + 31 + 33 + 50 + 53, 43 + 49 + 52 + 69 + 73, 44 + 47 + 65, and 77 + 85 + 110 + 111 + 115 + 116 + 117 depicted in Figure S13. However, congeners classified as non-Aroclor show a different trend, but it is also interesting that they only started reporting these congeners from 2006 (Figure S14). For example, PCB 11, and to a lesser extent PCB 68, shows an even more constant temporal trend than ΣPCB, where values from 2019 are very similar or higher than values from 2006. However, PCBs 35 and 67 show a clearer decline with time.

The application of the LME model to the individual locations, incorporating sites as a random effect along with water flow and temperature (when available), significantly improved model fitness and satisfied our assumptions; i.e., the residuals from the eq 1 show a normal distribution and have constant variance. This enhancement enabled the investigation of the half-lives of both ΣPCB and individual PCB congeners (Table S2). In general, the incorporation of a quadratic function to describe the flow did not improve the model’s performance.

Half-lives for ΣPCB were determined for the Anacostia, Fox, and Kalamazoo rivers and Chesapeake Bay, as well as New Bedford Harbor, meeting all specified criteria (i.e., statistically significant of the time coefficient from eq 1 and normality). Here, the Anacostia and Kalamazoo rivers as well as New Bedford Harbor yielded values of 2.8 (±1.0), 3.2 (±0.1), and 5 (±0.6) yr, respectively. Chesapeake Bay and the Fox River yielded higher values of 21 (±10) and 11 (±1.9) yr, respectively. We could only find one study of half-life calculations from empirical data for ΣPCB in water,^34^ which reported a value of 13 years. This half-life value was derived from the tributary data from the Lake Michigan Mass Balance Project.^23^ However, our LME approach did not yield a significant result using the tributary data. Zhang et al. used this value to develop a transport and fate model for Lake Michigan.

Only 38 individual or coeluting congeners from 11 locations yielded statistically significant half-lives, resulting in a total of 67 individual half-lives (see Table S2). Most of these half-lives (70%) were positive, indicating a decrease in the concentrations of individual congeners over time. The half-lives of congeners ranged from PCB 3 to PCB 209, with values between −16 ± 6 and 44 ± 19 yr^–1^. Among these findings, several are particularly noteworthy. For instance, at Portland Harbor, PCBs 6, 15, and 67 showed half-lives around −0.06 days, suggesting that their concentrations could double in less than 1.5 h. However, these results might be misleading due to the short time frame of sample collection (August 2018–January 2019), with samples from November and December 2018 being slightly higher, potentially skewing these calculations. On the other hand, PCBs 189 and 209 from the Passaic and Spokane rivers, respectively, yielded significant values in the range of −3 yr^–1^, where the data clearly suggest and increase with time. PCB 3 from the Passaic River yielded the lowest value, −16 ± 6 yr^–1^. Other examples included PCB 11 (−5 ± 2 yr^–1^) from the Fox River and PCB 67 (−0.1 ± 0.05 yr^–1^) from Portland Harbor. One possible explanation is that these locations are being impacted by non-Aroclor sources. Another finding was the location-specific nature of the half-lives. For instance, PCBs 5 + 8 yielded 9 ± 4 yr^–1^ in Chesapeake Bay and 4 ± 0.4 yr^–1^ in New Bedford Harbor. Caution is advised when half-life data are applied from one location to another. In many cases, our calculations were based on data from only a few years, which could lead to misleading conclusions. Furthermore, it was often impossible to determine statistically significant half-lives for all locations and congeners primarily because the original measurements were not designed for this type of analysis.

## Conclusions

**Figure 4:**
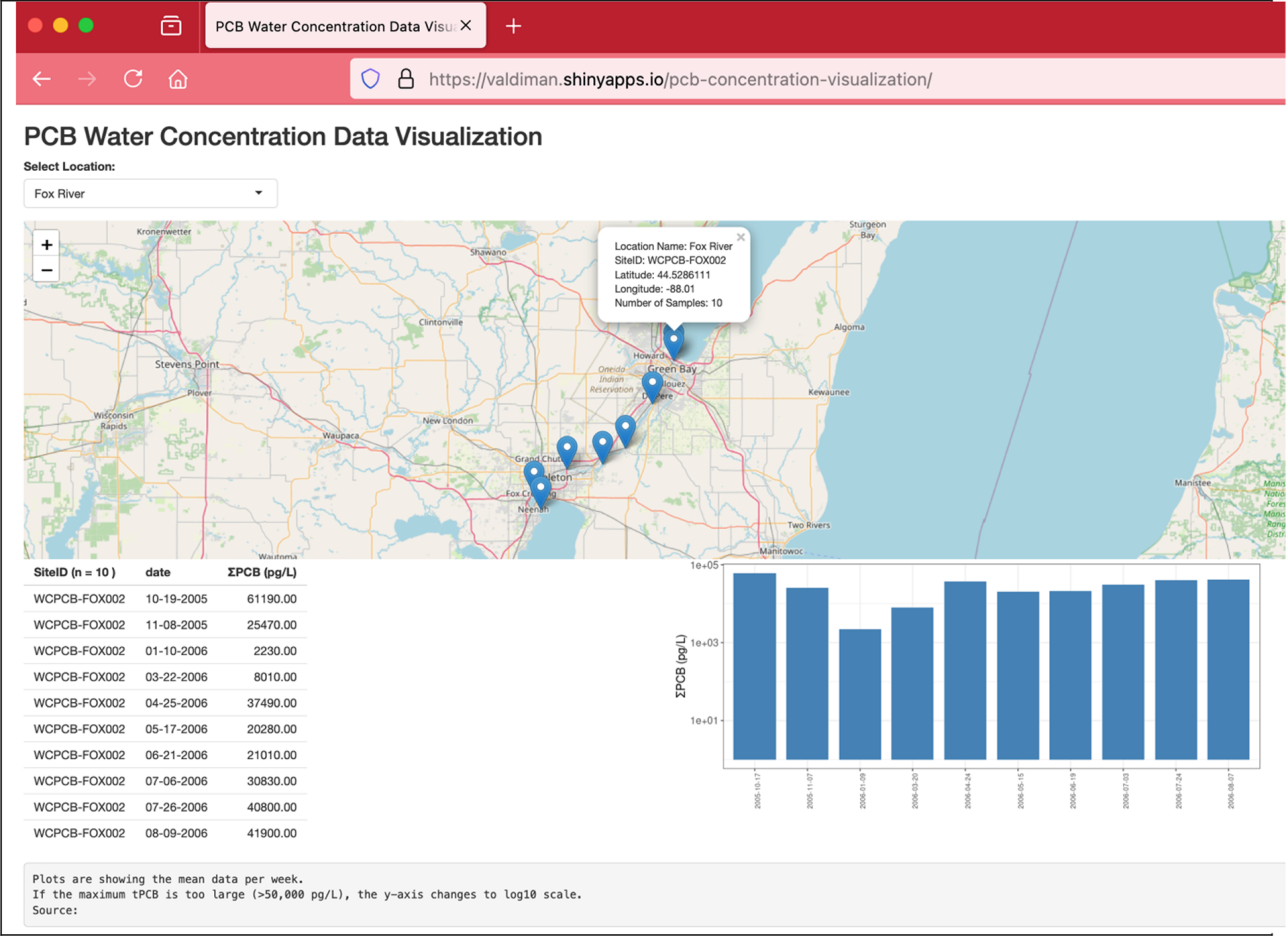
Interactive web-based map of ΣPCB in pg/L around the U.S. The screenshot shows data from the Fox River, WI, at one location. Web site is available at: https://valdiman.shinyapps.io/pcb-concentration-visualization/.

Obtaining dissolved PCB water concentration data proved challenging, even for PCB Superfund site locations. However, we successfully created a data set enabling investigation into spatial and temporal variability as well as the prediction of total and individual PCB concentrations in water. From this dataset, we calculated half-lives for individual PCBs and ΣPCB, revealing a general trend of decreasing concentrations over time at selected locations. Utilizing the Random Forest model, we can predict PCB water concentrations for most study locations by knowing the coordinates of the sampling location, sampling date, and relevant covariates such as season, flow, and temperature, if available, thus facilitating the estimation of dissolved PCB levels for future research, particularly for assessing PCB air–water exchange dynamics. This model approach, as demonstrated in this study, can also be applied to other locations, providing a flexible tool for analyzing PCB contamination across diverse aquatic systems. Additionally, the development of an interactive web-based map enhances our ability to explore sample locations, sample quantities, concentration ranges, and temporal trends (Figure 4). To put these data into a broader context, more sampling is needed in more isolated areas for background measurements as well as from water systems that flow through highly populated areas. Passive sampling could be a more feasible alternative to active sampling. Finally, the EPA or another agency or private group should consider creating a more comprehensive data set of PCB water concentrations across the country. This data set could build on what is presented here and be similar to those created for nitrate and PFAS.^35^

## Data Availability

The data underlying this study are openly available in Pangaea at 10.1594/PANGAEA.972705.^20^

## Supporting Information Available

Total PCB and individual PCB congeners water concentration maps, plots of occurrence and distribution of total PCB and individual congeners (PDF).

## Accession Codes

The R codes underlying this study are openly available in Zenodo at 10.5281/zenodo.13887687.^21^
