# Supplementary material for: Spatial and Temporal Analysis, and Machine Learning-Based Prediction of PCB Water Concentrations in U.S. Natural Water Systems
Source: ACS ES T Water. 2024 Dec 17;5(1):60–9. doi: 10.1021/acsestwater.4c00542 (PMC11731268; doi:10.1021/acsestwater.4c00542)
Supplement: Supplementary file 1 — ew4c00542_si_001.pdf [file ew4c00542_si_001.pdf]

Supporting Information: Spatial and Temporal Analysis, and Machine Learning-Based Prediction of PCB Water Concentrations in U.S. Natural Water Systems

Andres Martinez [a,*], Keri C. Hornbuckle [a], Michael P. Jones [b], and Brian D. Westra [c],

[a] Department of Civil & Environmental Engineering, IIHR-Hydroscience and Engineering, The University of Iowa, Iowa City, IA 52242, United States
[b] Department of Biostatistics, The University of Iowa, Iowa City, IA 52242, United States.
c University of Iowa Libraries, The University of Iowa, Iowa City, IA 52242, United States
* Email: andres-martinez@uiowa.edu

19 pages: 14 figures and 2 tables.

The final dataset is published open access at https://doi.org/10.1594/PANGAEA.972705 (Martinez 2024). Further, all the R codes created here to analyzed, model, generate the plots and maps are freely available at: https://doi.org/10.5281/zenodo.13887687 (Martinez 2024).

wdc

Filter | Cols: 1 – 50

	Source	SampleID	EPARegion	StateSampled	LocationName	SiteName	SiteID	SampleDate	Latitude	Longitude	Units	PhaseMeasured	EPAMethod	AroclorCongener	PCB1	PCB2	PCB3	PCB4.10
1	National Water Quality Monitoring Council. "Water Qu...	WCPCB-DMI001-20010711.1	R5	MI	DEQ (Michigan)	Au Sable River at Rea Rd	WCPCB-DMI001	2001-07-11	44.43612	-83.43417	pg/L	SurfaceWater	M1668	Congener	NA	NA	NA	15.68000
2	National Water Quality Monitoring Council. "Water Qu...	WCPCB-DMI001-20020710.1	R5	MI	DEQ (Michigan)	Au Sable River at Rea Rd	WCPCB-DMI001	2002-07-10	44.43612	-83.43417	pg/L	SurfaceWater	M1668	Congener	NA	NA	NA	62.66000
3	National Water Quality Monitoring Council. "Water Qu...	WCPCB-DMI001-20030624.1	R5	MI	DEQ (Michigan)	Au Sable River at Rea Rd	WCPCB-DMI001	2003-06-24	44.43612	-83.43417	pg/L	SurfaceWater	M1668	Congener	NA	NA	NA	NA
4	National Water Quality Monitoring Council. "Water Qu...	WCPCB-DMI001-20040331.1	R5	MI	DEQ (Michigan)	Au Sable River at Rea Rd	WCPCB-DMI001	2004-03-31	44.43612	-83.43417	pg/L	SurfaceWater	M1668	Congener	NA	NA	NA	NA
5	National Water Quality Monitoring Council. "Water Qu...	WCPCB-DMI001-20040427.1	R5	MI	DEQ (Michigan)	Au Sable River at Rea Rd	WCPCB-DMI001	2004-04-27	44.43612	-83.43417	pg/L	SurfaceWater	M1668	Congener	NA	NA	NA	NA
6	National Water Quality Monitoring Council. "Water Qu...	WCPCB-DMI001-20040512.1	R5	MI	DEQ (Michigan)	Au Sable River at Rea Rd	WCPCB-DMI001	2004-05-12	44.43612	-83.43417	pg/L	SurfaceWater	M1668	Congener	NA	NA	NA	NA
7	National Water Quality Monitoring Council. "Water Qu...	WCPCB-DMI001-20040519.1	R5	MI	DEQ (Michigan)	Au Sable River at Rea Rd	WCPCB-DMI001	2004-05-19	44.43612	-83.43417	pg/L	SurfaceWater	M1668	Congener	NA	NA	NA	NA
8	National Water Quality Monitoring Council. "Water Qu...	WCPCB-DMI001-20040608.1	R5	MI	DEQ (Michigan)	Au Sable River at Rea Rd	WCPCB-DMI001	2004-06-08	44.43612	-83.43417	pg/L	SurfaceWater	M1668	Congener	NA	NA	NA	NA
9	National Water Quality Monitoring Council. "Water Qu...	WCPCB-DMI001-20040713.1	R5	MI	DEQ (Michigan)	Au Sable River at Rea Rd	WCPCB-DMI001	2004-07-13	44.43612	-83.43417	pg/L	SurfaceWater	M1668	Congener	NA	NA	NA	NA
10	National Water Quality Monitoring Council. "Water Qu...	WCPCB-DMI001-20040729.1	R5	MI	DEQ (Michigan)	Au Sable River at Rea Rd	WCPCB-DMI001	2004-07-29	44.43612	-83.43417	pg/L	SurfaceWater	M1668	Congener	NA	NA	NA	37.03000
11	National Water Quality Monitoring Council. "Water Qu...	WCPCB-DMI001-20040825.1	R5	MI	DEQ (Michigan)	Au Sable River at Rea Rd	WCPCB-DMI001	2004-08-25	44.43612	-83.43417	pg/L	SurfaceWater	M1668	Congener	NA	NA	NA	NA
12	National Water Quality Monitoring Council. "Water Qu...	WCPCB-DMI001-20040914.1	R5	MI	DEQ (Michigan)	Au Sable River at Rea Rd	WCPCB-DMI001	2004-09-14	44.43612	-83.43417	pg/L	SurfaceWater	M1668	Congener	NA	NA	NA	NA
13	National Water Quality Monitoring Council. "Water Qu...	WCPCB-DMI001-20041013.1	R5	MI	DEQ (Michigan)	Au Sable River at Rea Rd	WCPCB-DMI001	2004-10-13	44.43612	-83.43417	pg/L	SurfaceWater	M1668	Congener	NA	NA	NA	NA
14	National Water Quality Monitoring Council. "Water Qu...	WCPCB-DMI001-20041027.1	R5	MI	DEQ (Michigan)	Au Sable River at Rea Rd	WCPCB-DMI001	2004-10-27	44.43612	-83.43417	pg/L	SurfaceWater	M1668	Congener	NA	NA	NA	NA
15	National Water Quality Monitoring Council. "Water Qu...	WCPCB-DMI001-20041123.1	R5	MI	DEQ (Michigan)	Au Sable River at Rea Rd	WCPCB-DMI001	2004-11-23	44.43612	-83.43417	pg/L	SurfaceWater	M1668	Congener	NA	NA	NA	NA
16	National Water Quality Monitoring Council. "Water Qu...	WCPCB-DMI002-20010809.1	R5	MI	DEQ (Michigan)	Au Train Bay Scott Falls	WCPCB-DMI002	2001-08-09	46.43728	-86.81744	pg/L	SurfaceWater	M1668	Congener	NA	NA	NA	80.32000
17	National Water Quality Monitoring Council. "Water Qu...	WCPCB-DMI002-20020730.1	R5	MI	DEQ (Michigan)	Au Train Bay Scott Falls	WCPCB-DMI002	2002-07-30	46.43728	-86.81744	pg/L	SurfaceWater	M1668	Congener	NA	NA	NA	109.99000
18	National Water Quality Monitoring Council. "Water Qu...	WCPCB-DMI002-20030610.1	R5	MI	DEQ (Michigan)	Au Train Bay Scott Falls	WCPCB-DMI002	2003-06-10	46.43728	-86.81744	pg/L	SurfaceWater	M1668	Congener	NA	NA	NA	110.65000
19	National Water Quality Monitoring Council. "Water Qu...	WCPCB-DMI002-20050321.1	R5	MI	DEQ (Michigan)	Au Train Bay Scott Falls	WCPCB-DMI002	2005-03-21	46.43728	-86.81744	pg/L	SurfaceWater	M1668	Congener	NA	NA	NA	NA
20	National Water Quality Monitoring Council. "Water Qu...	WCPCB-DMI002-20050504.1	R5	MI	DEQ (Michigan)	Au Train Bay Scott Falls	WCPCB-DMI002	2005-05-04	46.43728	-86.81744	pg/L	SurfaceWater	M1668	Congener	NA	NA	NA	NA
21	National Water Quality Monitoring Council. "Water Qu...	WCPCB-DMI002-20050601.1	R5	MI	DEQ (Michigan)	Au Train Bay Scott Falls	WCPCB-DMI002	2005-06-01	46.43728	-86.81744	pg/L	SurfaceWater	M1668	Congener	NA	NA	NA	NA
22	National Water Quality Monitoring Council. "Water Qu...	WCPCB-DMI002-20050628.1	R5	MI	DEQ (Michigan)	Au Train Bay Scott Falls	WCPCB-DMI002	2005-06-28	46.43728	-86.81744	pg/L	SurfaceWater	M1668	Congener	NA	NA	NA	NA
23	National Water Quality Monitoring Council. "Water Qu...	WCPCB-DMI002-20050803.1	R5	MI	DEQ (Michigan)	Au Train Bay Scott Falls	WCPCB-DMI002	2005-08-03	46.43728	-86.81744	pg/L	SurfaceWater	M1668	Congener	NA	NA	NA	122.51000
24	National Water Quality Monitoring Council. "Water Qu...	WCPCB-DMI002-20050824.1	R5	MI	DEQ (Michigan)	Au Train Bay Scott Falls	WCPCB-DMI002	2005-08-24	46.43728	-86.81744	pg/L	SurfaceWater	M1668	Congener	NA	NA	NA	115.09000
25	National Water Quality Monitoring Council. "Water Qu...	WCPCB-DMI002-20050914.1	R5	MI	DEQ (Michigan)	Au Train Bay Scott Falls	WCPCB-DMI002	2005-09-14	46.43728	-86.81744	pg/L	SurfaceWater	M1668	Congener	NA	NA	NA	76.05000
26	National Water Quality Monitoring Council. "Water Qu...	WCPCB-DMI002-20050927.1	R5	MI	DEQ (Michigan)	Au Train Bay Scott Falls	WCPCB-DMI002	2005-09-27	46.43728	-86.81744	pg/L	SurfaceWater	M1668	Congener	NA	NA	NA	108.33000
27	National Water Quality Monitoring Council. "Water Qu...	WCPCB-DMI002-20051018.1	R5	MI	DEQ (Michigan)	Au Train Bay Scott Falls	WCPCB-DMI002	2005-10-18	46.43728	-86.81744	pg/L	SurfaceWater	M1668	Congener	NA	NA	NA	NA
28	National Water Quality Monitoring Council. "Water Qu...	WCPCB-DMI002-20051102.1	R5	MI	DEQ (Michigan)	Au Train Bay Scott Falls	WCPCB-DMI002	2005-11-02	46.43728	-86.81744	pg/L	SurfaceWater	M1668	Congener	NA	NA	NA	62.92000
29	National Water Quality Monitoring Council. "Water Qu...	WCPCB-DMI002-20051122.1	R5	MI	DEQ (Michigan)	Au Train Bay Scott Falls	WCPCB-DMI002	2005-11-22	46.43728	-86.81744	pg/L	SurfaceWater	M1668	Congener	NA	NA	NA	40.98000
30	National Water Quality Monitoring Council. "Water Qu...	WCPCB-DMI003-20040701.1	R5	MI	DEQ (Michigan)	Bellamy Creek Bluewater HWY	WCPCB-DMI003	2004-07-01	42.97918	-85.11105	pg/L	SurfaceWater	M1668	Congener	NA	NA	NA	44.52000
31	National Water Quality Monitoring Council. "Water Qu...	WCPCB-DMI004-20020626.1	R5	MI	DEQ (Michigan)	Big S Branch Pere Marquette River N Dickinson Ave	WCPCB-DMI004	2002-06-26	43.78435	-86.01869	pg/L	SurfaceWater	M1668	Congener	NA	NA	NA	77.77000

PCB204	PCB206	PCB207	PCB209	A1016	A1221	A1232	A1242	A1248	A1254	A1260	tPCB
NA	NA	NA	NA	NA	NA	NA	NA	NA	NA	NA	116.25
NA	NA	NA	NA	NA	NA	NA	NA	NA	NA	NA	660.39
NA	NA	NA	NA	NA	NA	NA	NA	NA	NA	NA	148.72
NA	NA	NA	NA	NA	NA	NA	NA	NA	NA	NA	37.47
NA	NA	NA	NA	NA	NA	NA	NA	NA	NA	NA	39.77
NA	NA	NA	NA	NA	NA	NA	NA	NA	NA	NA	107.49
NA	NA	NA	NA	NA	NA	NA	NA	NA	NA	NA	38.61
NA	NA	NA	NA	NA	NA	NA	NA	NA	NA	NA	106.43
NA	NA	NA	NA	NA	NA	NA	NA	NA	NA	NA	67.01
NA	NA	NA	NA	NA	NA	NA	NA	NA	NA	NA	231.33
NA	NA	NA	NA	NA	NA	NA	NA	NA	NA	NA	140.30
NA	NA	NA	NA	NA	NA	NA	NA	NA	NA	NA	59.58
NA	NA	NA	NA	NA	NA	NA	NA	NA	NA	NA	99.15
NA	NA	NA	NA	NA	NA	NA	NA	NA	NA	NA	17.05
NA	NA	NA	NA	NA	NA	NA	NA	NA	NA	NA	71.09
NA	NA	NA	NA	NA	NA	NA	NA	NA	NA	NA	8180.56
NA	NA	NA	NA	NA	NA	NA	NA	NA	NA	NA	9469.48
NA	NA	NA	NA	NA	NA	NA	NA	NA	NA	NA	7882.91
NA	NA	NA	NA	NA	NA	NA	NA	NA	NA	NA	2698.31
NA	NA	NA	NA	NA	NA	NA	NA	NA	NA	NA	6067.13
NA	NA	NA	NA	NA	NA	NA	NA	NA	NA	NA	7955.78
NA	NA	NA	NA	NA	NA	NA	NA	NA	NA	NA	7326.79
NA	NA	NA	NA	NA	NA	NA	NA	NA	NA	NA	6595.09
NA	NA	NA	NA	NA	NA	NA	NA	NA	NA	NA	6006.43

Figure S1 Screenshot of the PCB water concentration dataset generated in R. NA refers to value not reported.

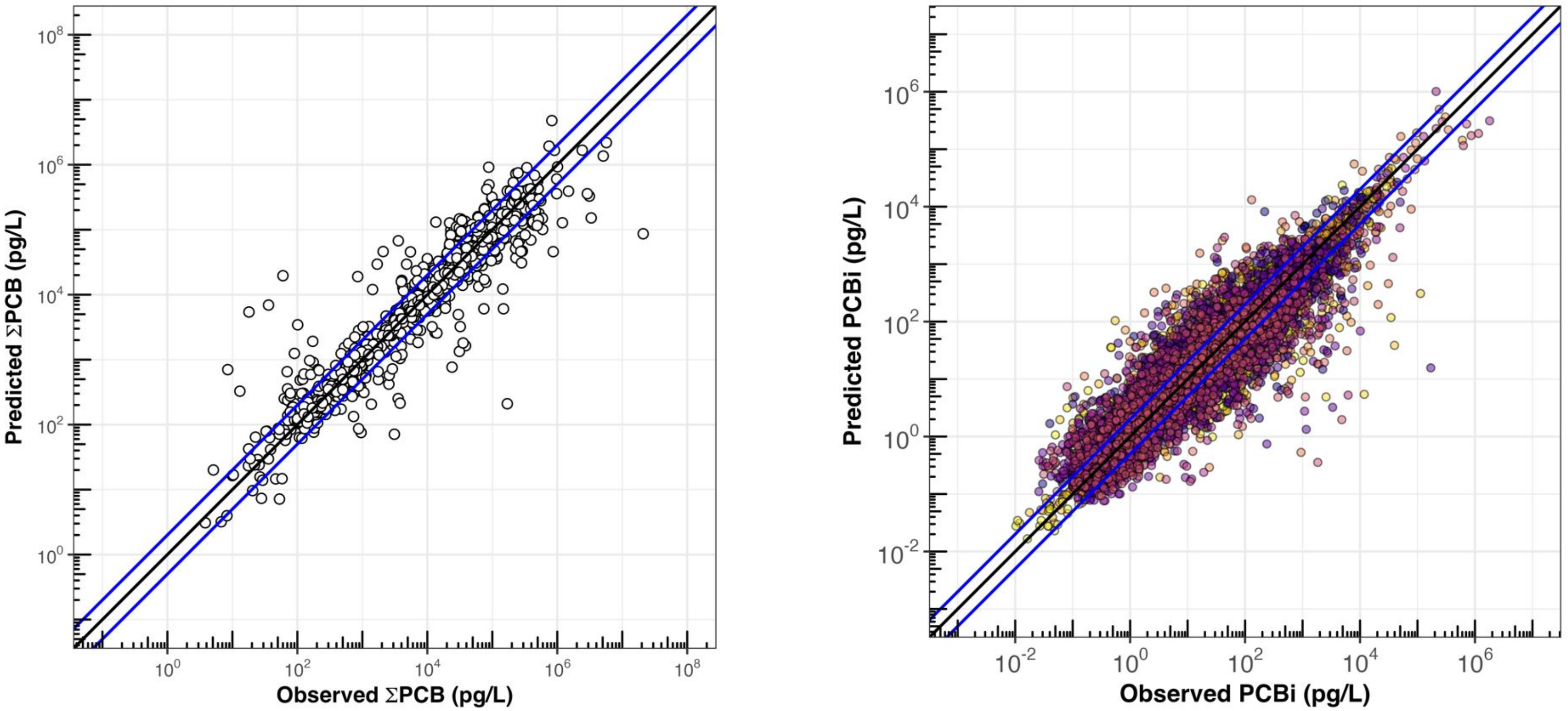

Figure S2 Random Forest predictions vs observations. Left plot shows results for ΣPCB (RMSE of 0.38, Pearson correlation coefficient of 0.94 and within a factor of 2, 75% of the time). The Random Forest model optimization for ΣPCB yielded the following parameters: number of trees = 5000, number of variables randomly sampled as candidates at each split = 3, split rule for decision trees = variance reduction, and minimum size of terminal nodes in the trees = 5. Right plot represents 61 individual PCB congeners, resulting in an average RMSE of 0.35 (± 0.04), average of the Pearson correlation coefficient of 0.93 (± 0.02), and average of the factor of 2, 76% (± 6%) of the time. The Random Forest model optimization for individual PCB yielded the following range parameters: number of trees = 500 - 5000, number of variables randomly sampled as candidates at each split = 2 - 3, split rule for decision trees = variance reduction, and minimum size of terminal nodes in the trees = 3-10. Black line represents the 1:1 line, and the blue lines represent the 1:2 and 2:1 lines (i.e., factor of 2).

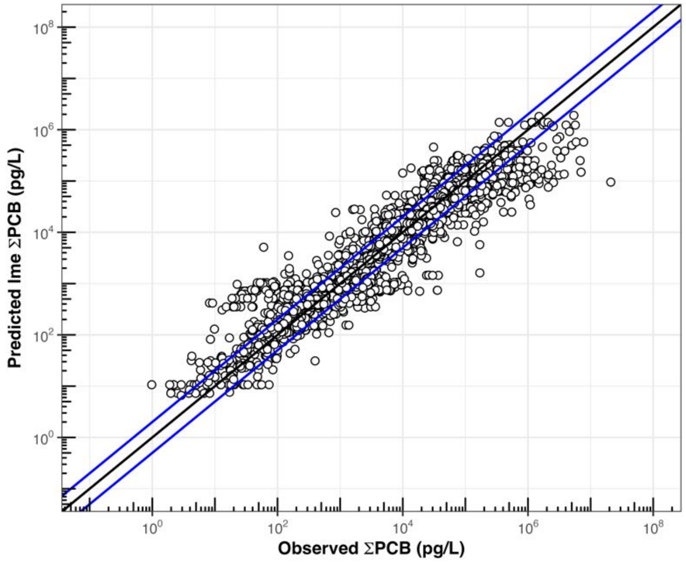

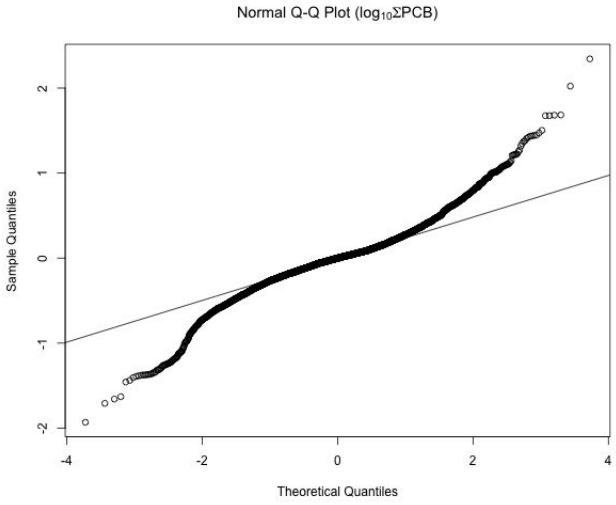

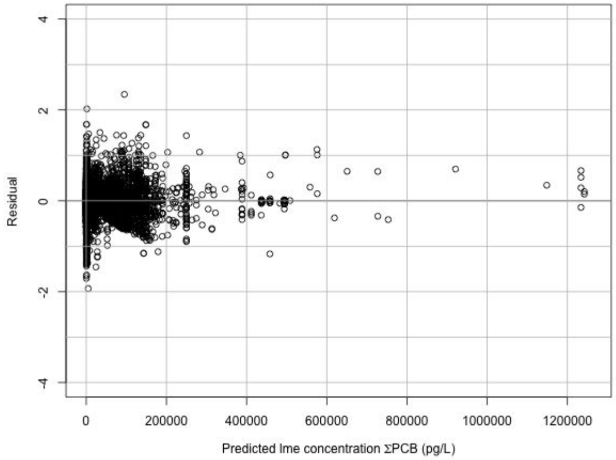

Figure S3 Left top panel shows the LME predictions vs observations of ΣPCB in pg/L for all samples ($R^2 = 0.88$), where the black line represents the 1:1 line, and the blue lines represent the 1:2 and 2:1 lines (i.e., factor of 2). Right top panel shows the Q-Q plot of the residuals and bottom left panel shows the residuals vs the predictions. Aroclor and samples with individual congeners are included (n = 5132).

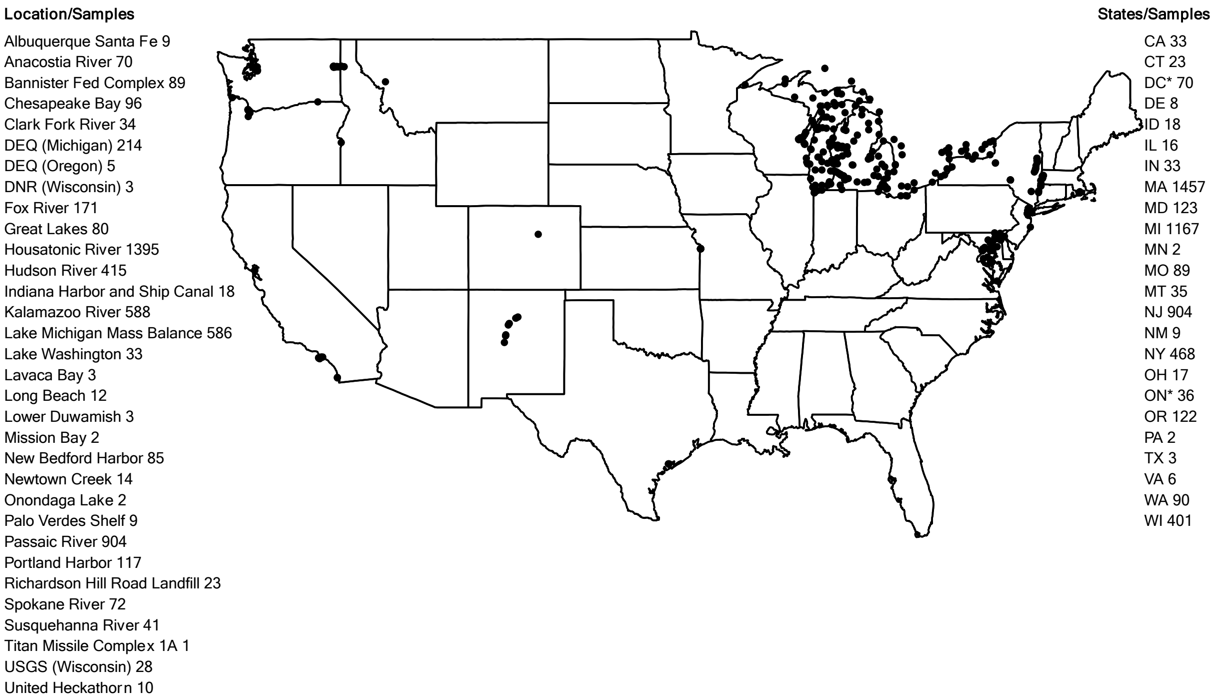

Figure S4 USA map showing sampling locations, with the number of samples per state. DC and ON are District of Columbia and Ontario, Canada, respectively.

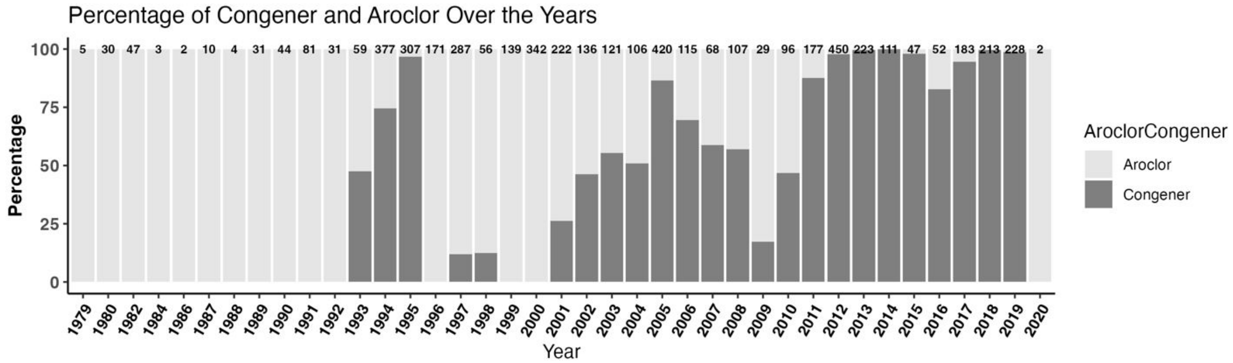

Figure S5 Percentages of samples analyzed using an Aroclor method or a congener method. Numbers on each bar represent the total number of samples per year.

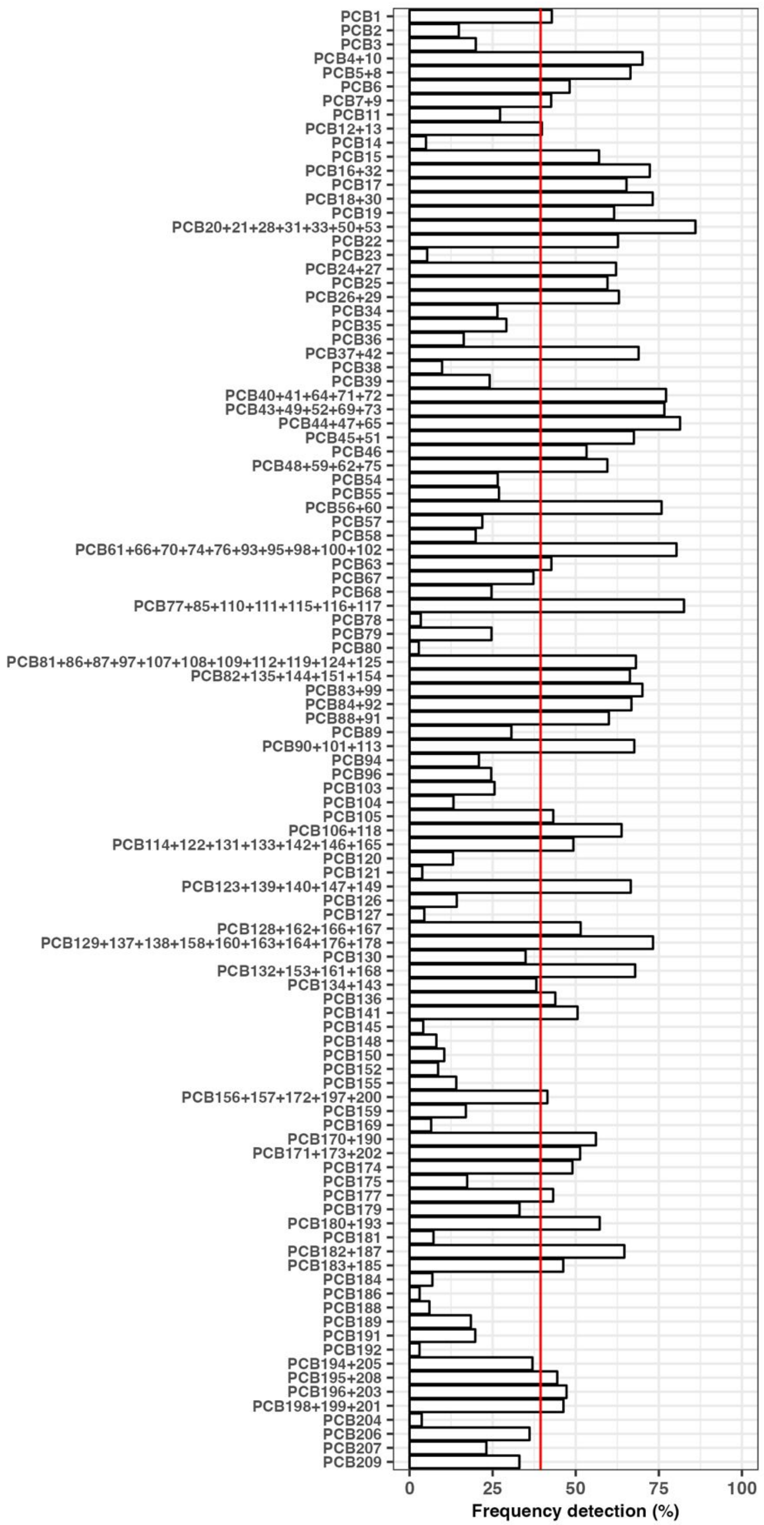

Figure S6 Frequency detection of individual PCB congeners from data that reported individual congeners (n = 3116). Red line represents the frequency average of all PCB congeners (41%).

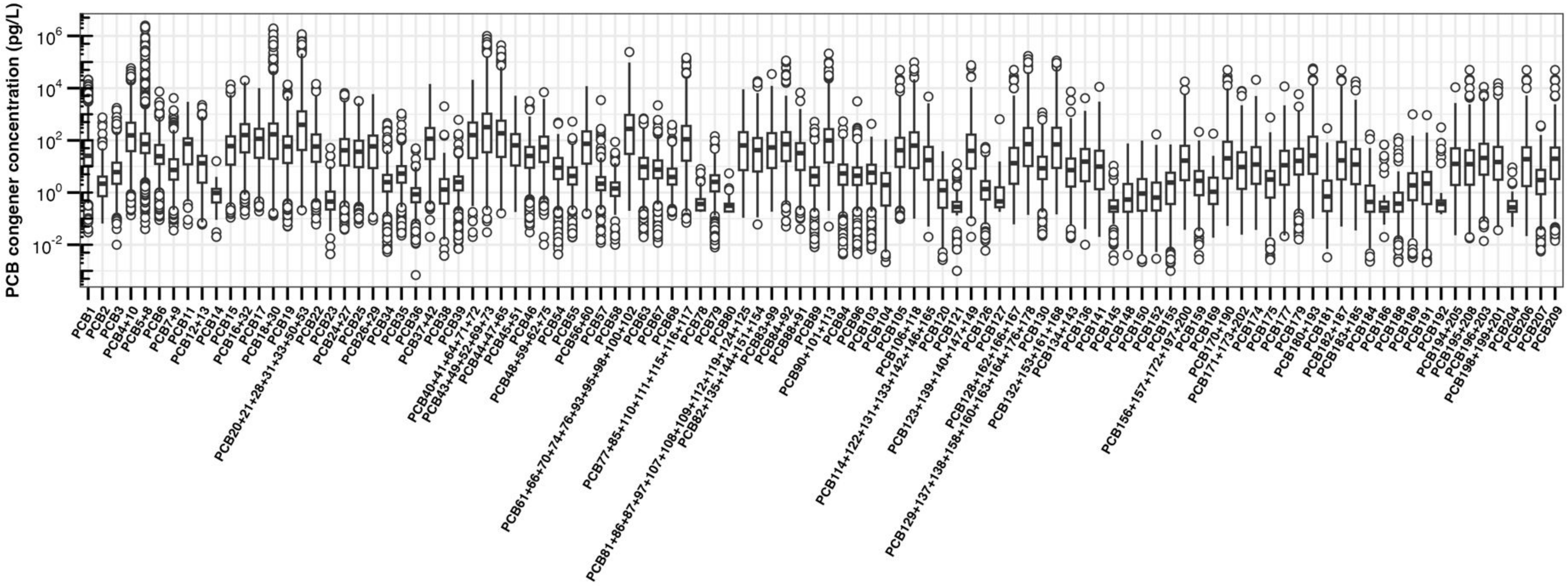

Figure S7 Summary of water samples of individual PCB congeners (n = 104) from data that only reported individual PCB congeners (n = 3110).

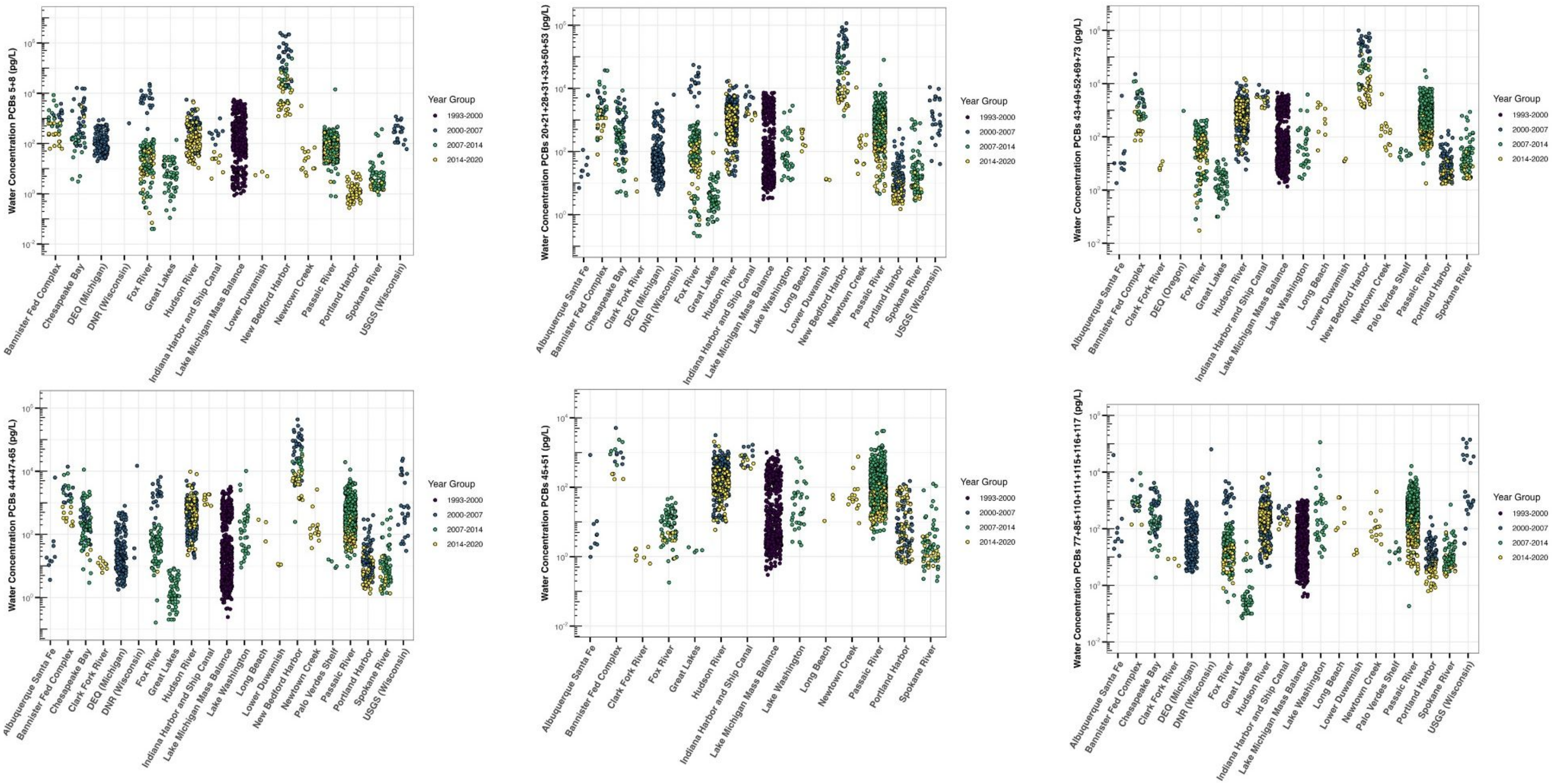

Figure S8 Selected Aroclor PCB coeluting congeners including PCBs 5+8, 20+21+28+31+33+50+53, 43+49+52+69+73, 44+47+65, 45+51 and 77+85+110+111+115+116+117 water concentrations (pg/L) for selected locations. Color describes the PCB over 6-year periods.

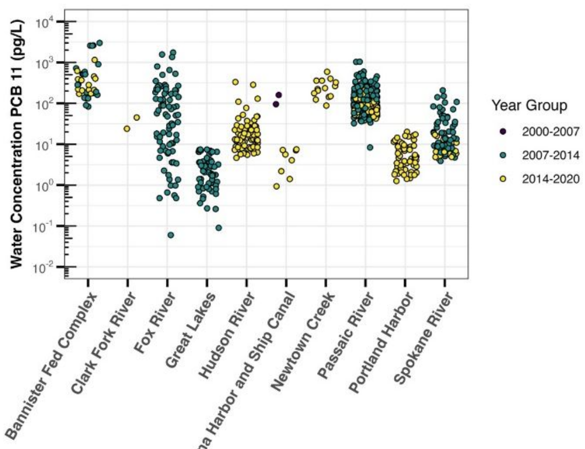

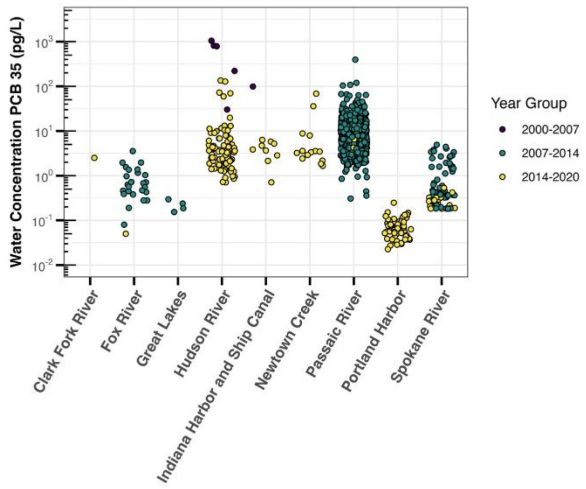

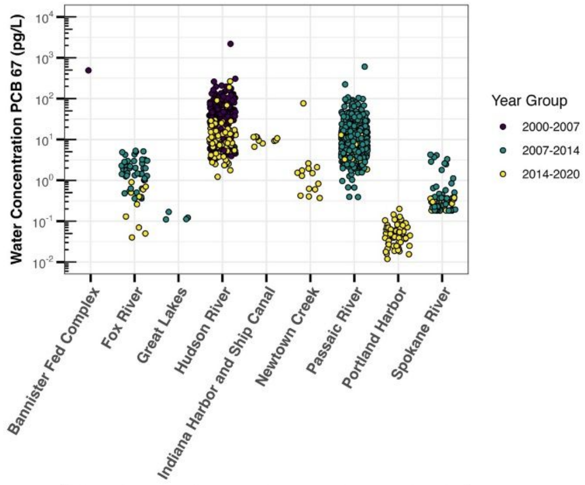

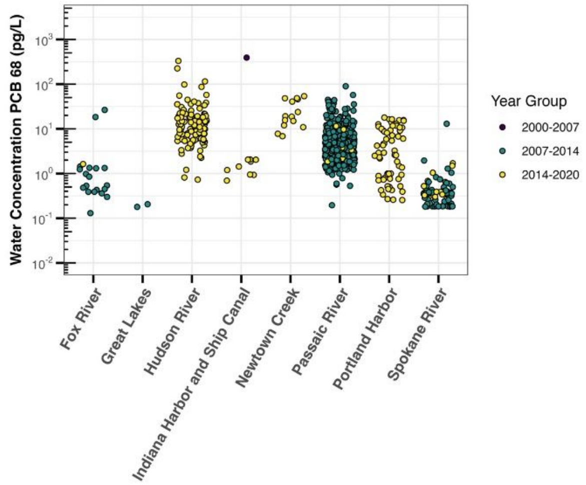

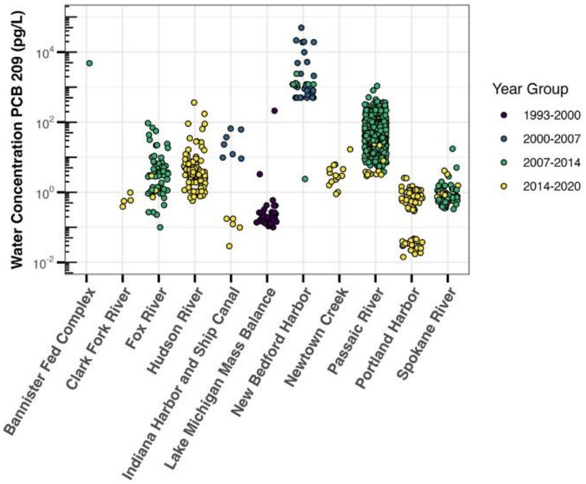

Figure S9 Selected individual PCB congeners including PCBs 11, 35, 67, 68 and 209 in water concentrations (pg/L) for selected locations. All these congeners are classified as non-Aroclor congeners. Color describes the PCB over 6-year periods.

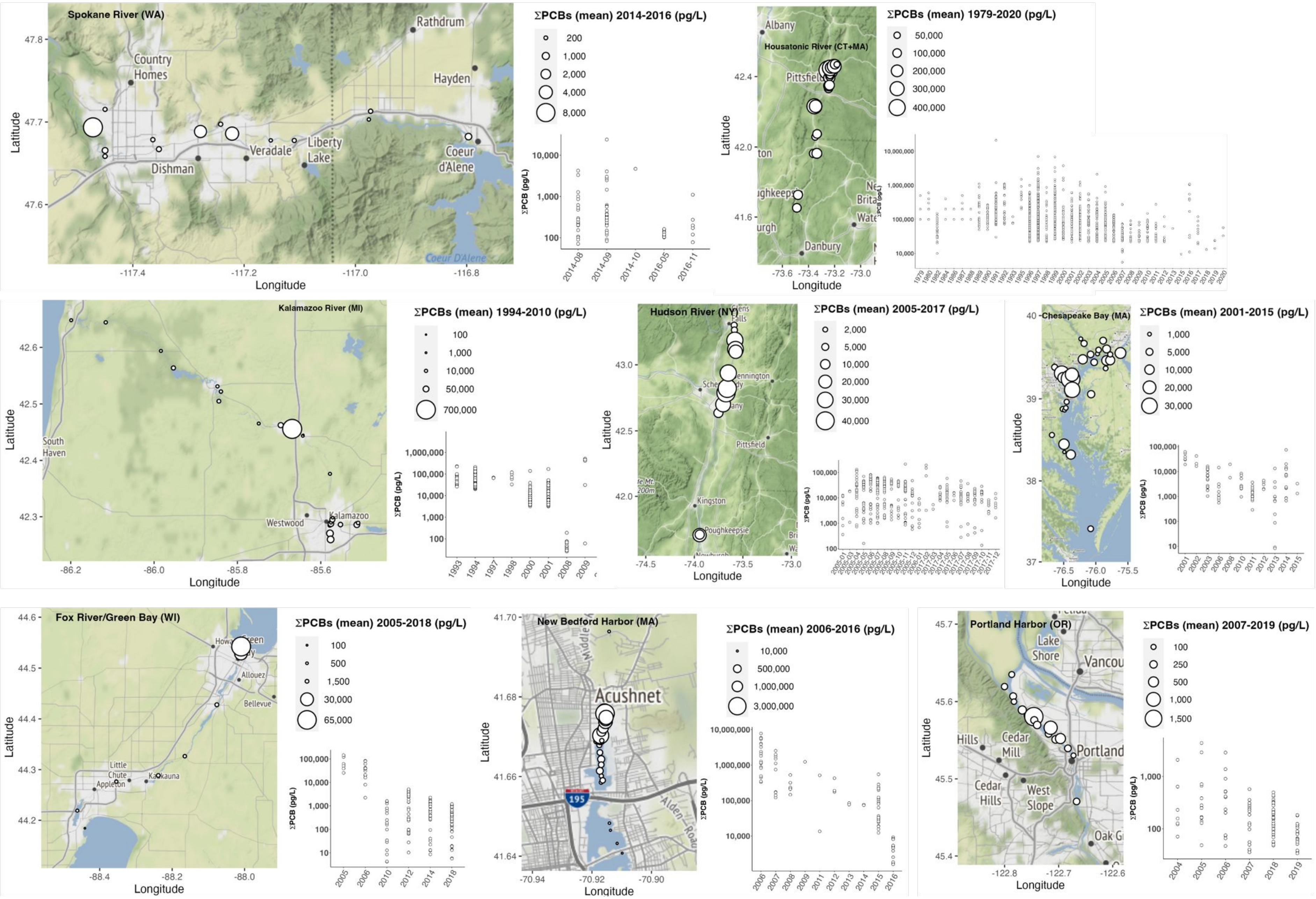

Figure S10 Spatial and temporal distribution of total PCBs for eight PCB-contaminated sites in the USA. Maps illustrate the average concentration per site, while the temporal plots show all the data.

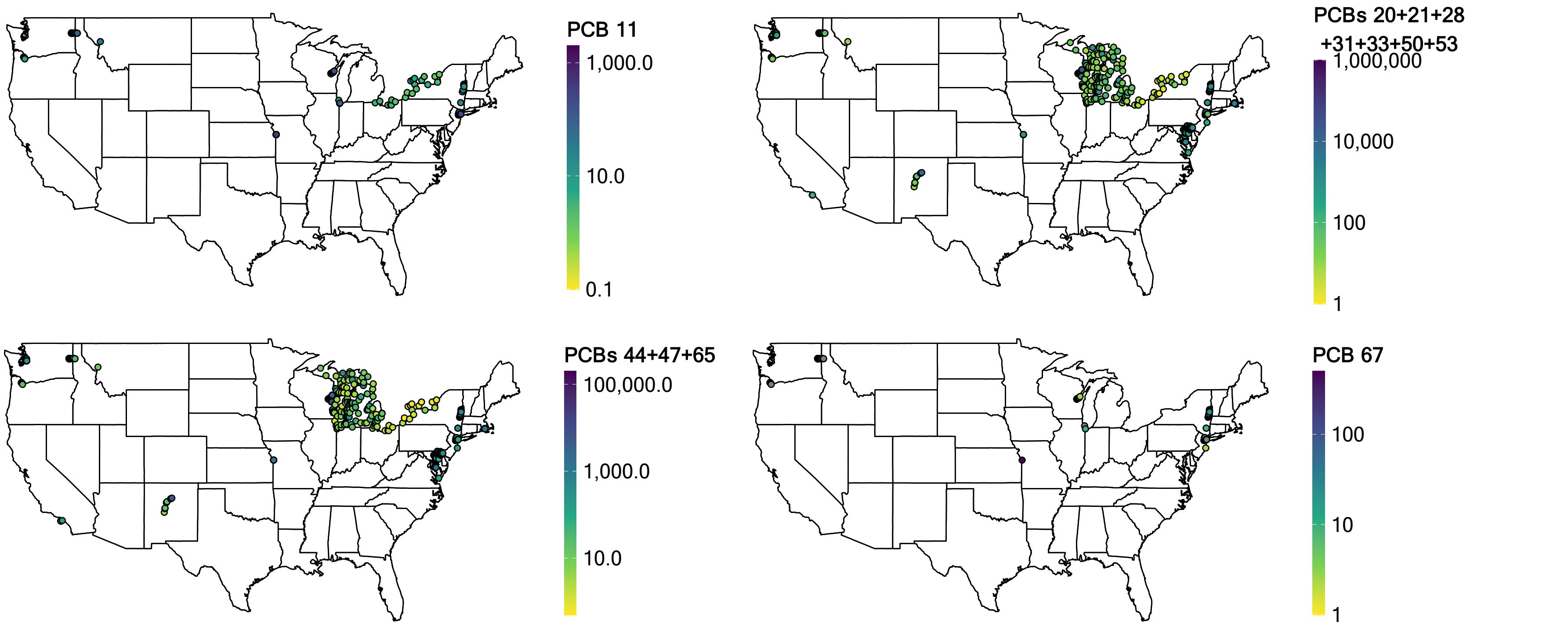

Figure S11 Spatial distribution of selected individual PCB congeners or coeluting congeners including PCB 11, PCBs 20+21+28+31+33+50+53, PCBs 44+47+65 and PCB 67 water concentrations (pg/L). PCBs 20+21+28+31+33+50+53 and 44+47+65 are classified as Aroclor congeners, while 11 and 67 are classified as non-Aroclor congeners. It's important to note that the location of PCB 67, with a concentration of approximately 400 pg/L, represents only one sample from the Bannister Federal Complex in 2004. Additionally, a few locations are not shown here, as depicted in Fig. S4, because these locations were analyzed using an Aroclor method.

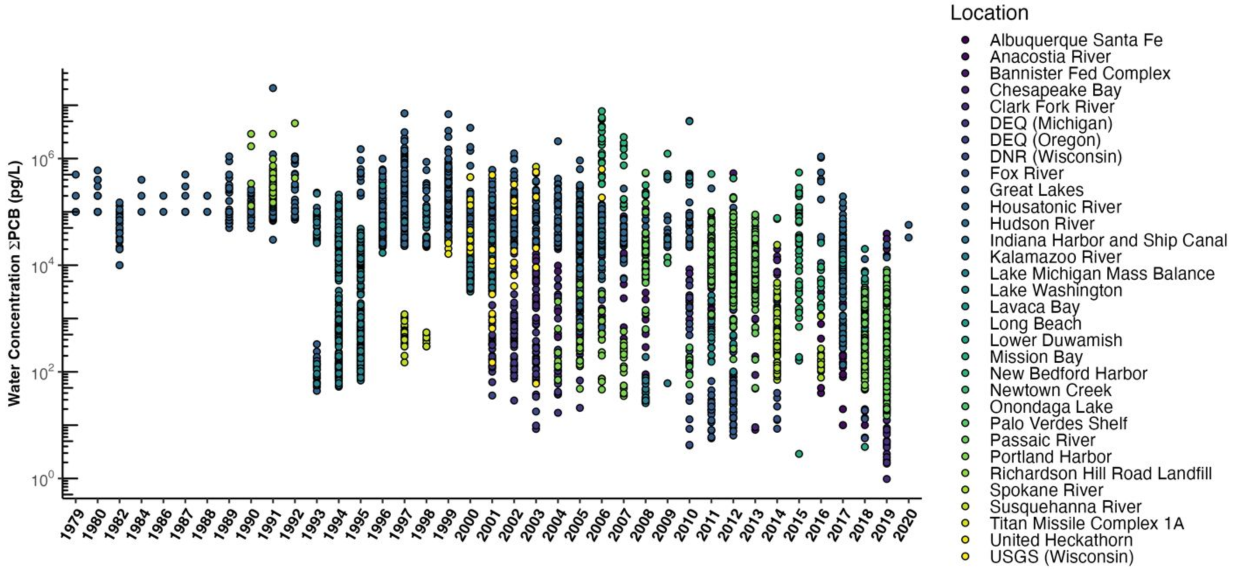

Figure S12 Total PCB water concentration versus time in years. Aroclor and individual congeners data were used (n = 5138). Locations are illustrated in different colors.

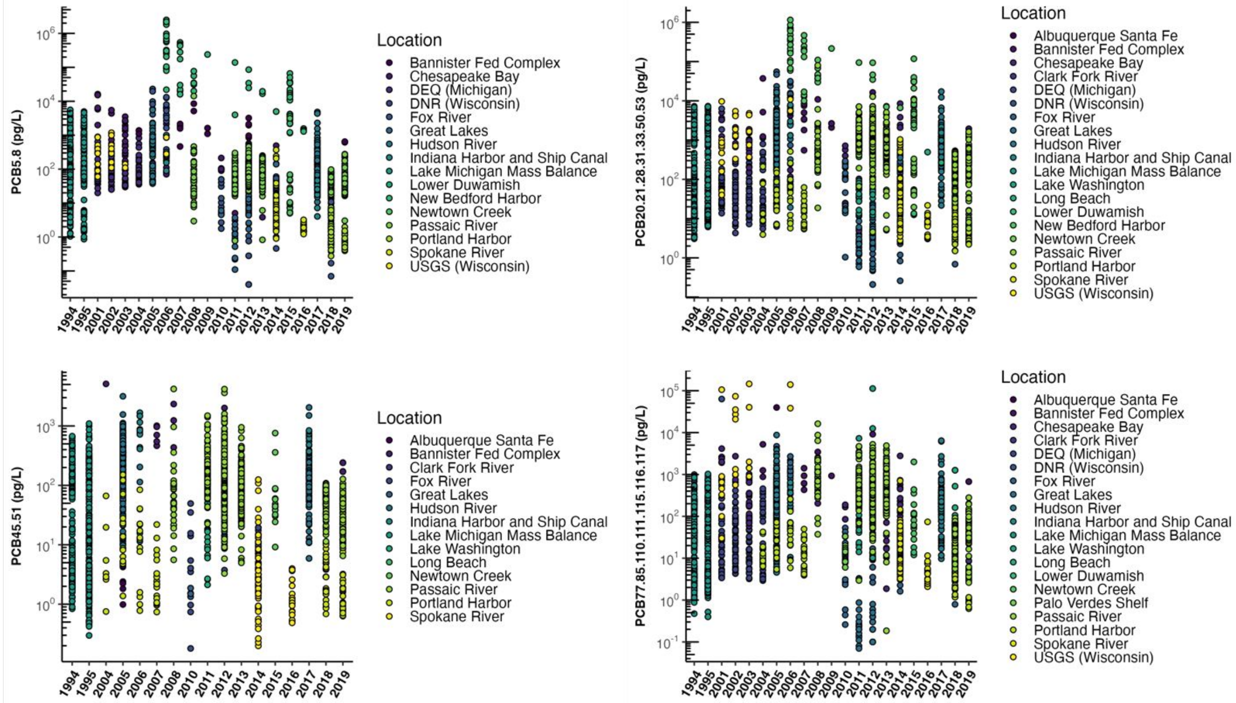

Figure S13 Selected PCB coeluting congeners including PCBs 5+8, 20+21+28+31+33+50+53, 43+49+52+69+73, 44+47+65 and 77+85+110+111+115+116+117 water concentrations (pg/L) versus time in year. These congeners can be classified as Aroclor congeners. Locations are illustrated in different colors.

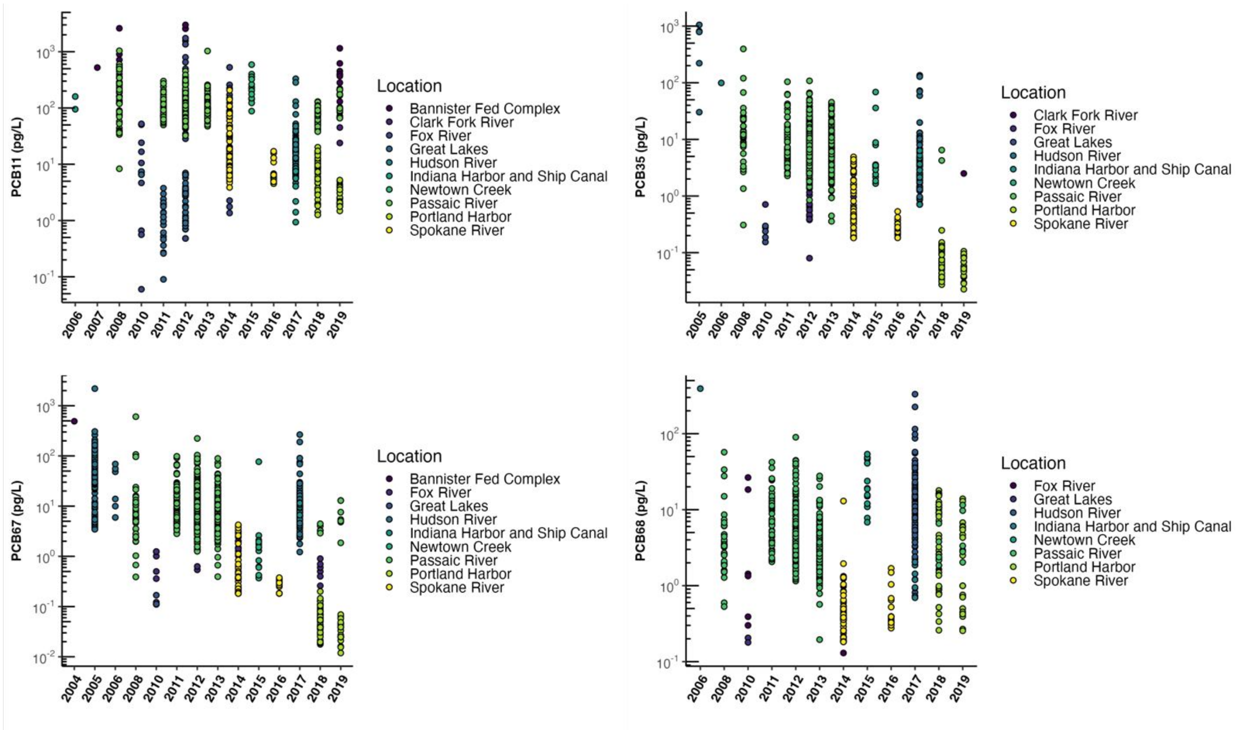

Figure S14 Selected non-Aroclor individual PCB congeners including PCBs 11, 35, 67, 68 and 209 in water concentrations (pg/L) versus time in year. These congeners can be classified as non-Aroclor congeners. Locations are illustrated in different colors.

Table S1 List of the 104 individual and coeluting PCB congeners.

Congener	Congener	Congener
PCB1	PCB56+60	PCB136
PCB2	PCB57	PCB141
PCB3	PCB58	PCB145
PCB4+10	PCB61+66+70+74+76+93+95+98+100+102	PCB148
PCB5+8	PCB63	PCB150
PCB6	PCB67	PCB152
PCB7+9	PCB68	PCB155
PCB11	PCB77+85+110+111+115+116+117	PCB156+157+172+197+200
PCB12+13	PCB78	PCB159
PCB14	PCB79	PCB169
PCB15	PCB80	PCB170+190
PCB16+32	PCB81+86+87+97+107+108+109+112+119+124+125	PCB171+173+202
PCB17	PCB82+135+144+151+154	PCB174
PCB18+30	PCB83+99	PCB175
PCB19	PCB84+92	PCB177
PCB20+21+28+31+33+50+53	PCB88+91	PCB179
PCB22	PCB89	PCB180+193
PCB23	PCB90+101+113	PCB181
PCB24+27	PCB94	PCB182+187
PCB25	PCB96	PCB183+185
PCB26+29	PCB103	PCB184
PCB34	PCB104	PCB186
PCB35	PCB105	PCB188
PCB36	PCB106+118	PCB189
PCB37+42	PCB114+122+131+133+142+146+165	PCB191
PCB38	PCB120	PCB192
PCB39	PCB121	PCB194+205
PCB40+71+64+71+72	PCB123+139+140+147+149	PCB195+208
PCB43+49+52+69+73	PCB126	PCB196+203
PCB44+47+65	PCB127	PCB198+199+201
PCB45+51	PCB128+162+166+167	PCB204
PCB46	PCB129+137+138+158+160+163+164+176+178	PCB206
PCB48+59+62+75	PCB130	PCB207
PCB54	PCB132+153+161+168	PCB209
PCB55	PCB134+143	

Table S2 Half-lives ($t_{0.5}$) for ΣPCB and individual PCB congeners. Half-lives were computed using the Linear Mixed-Effects Model. Q indicates that the flow was represented as a quadratic function in the LME model. Only statistically significant time coefficients from Eq. 1 are included here to compute $t_{0.5}$, as well as the residuals from the model showed a normal distribution, as confirmed by a Q-Q plot, and second, the residuals displayed a constant variance. In the table, n denotes the number of samples used in the analysis.

Location	Covariates	ΣPCB / Congeners	$t_{0.5}$	SE ($t_{0.5}$)	R^2	RMSE	Factor2
Anacostia River (n = 70)	Time, flow, water temperature, season, site (random)	ΣPCB	2.83	0.99	0.96	0.13	97.1
Chesapeake Bay (n = 96)	Time, water temperature, season, site (random)	ΣPCB	20.79	10.45	0.48	0.43	56.7
Fox River [1] (n = 171)	Time, flow, water temperature, season, site (random)	ΣPCB	11.07	1.91	0.78	0.22	85.3
Kalamazoo River [2] (n = 556)	Time, flow, season, site (random)	ΣPCB	3.25	0.11	0.97	0.2	84.9
New Bedford Harbor (n = 85)	Time, season, site (random)	ΣPCB	5.1	0.6	0.89	0.2	81.2
Chesapeake Bay (n = 80)	Time, water temperature, season, site (random)	PCB20+21+28+31+33+50+53	15.75	7.63	0.41	0.89	55.4
Chesapeake Bay (n = 81)		PCB129+137+138+158+160+163+164+176+178	14.92	5.24	0.58	0.72	62.7
Chesapeake Bay (n = 65)		PCB77+85+110+111+115+116+117	11.84	3.51	0.55	0.77	55.9
Chesapeake Bay (n = 51)		PCB5+8	9.18	3.56	0.66	1.03	53.3
Chesapeake Bay (n = 34)		PCB81+86+87+97+107+108+109+112+119+124+125	6.97	2.92	0.88	1.04	83.3
Chesapeake Bay (n = 80)		PCB56+60	6.83	1.52	0.69	1.08	52.7
Chesapeake Bay (n = 32)		PCB156+157+172+197+200	5.96	2.35	0.64	0.6	76.9
Chesapeake Bay (n = 35)		PCB88+91	2.19	0.38	0.6	0.71	65.7
DEQ MI (n = 180)	Time, season, site (random)	PCB61+66+70+74+76+93+95+98+100+102	16.0	7.63	0.78	0.71	86.7
DEQ MI (n = 202)		PCB20+21+28+31+33+50+53	12.06	4.08	0.78	0.52	83.2
Fox River [1] (n = 101)		PCB83+99	18.27	5.9	0.58	0.31	80.2
Fox River [1] (n = 101)		PCB17	14.35	2.98	0.81	0.2	87.1
Fox River [1] (n = 101)		PCB25	11.8	1.87	0.83	0.37	87.1

Fox River [1] (n = 102)		PCB20+21+28+31+33+50 +53	11.3	1.63	0.85	0.18	91.2
Fox River [1] (n = 102)		PCB18+30	10.81	1.39	0.88	0.16	92.2
Fox River [1] (n = 91)		PCB105	8.24	1.69	0.55	0.58	71.4
Fox River [1] (n = 65)		PCB177	7.35	1.74	0.56	0.65	70.8
Fox River [1] (n = 30)		PCB195+208	4.83	1.45	0.76	0.64	80.0
Fox River [1] (n = 47)		PCB67	4.14	0.51	0.84	0.59	85.1
Fox River [1] (n = 33)		PCB128+162+166+167	-9.87	2.34	0.91	0.53	100.0
Fox River Q [1] (n = 101)	Time, flow, water temperature, season, site (random)	PCB83+99	22.11	8.44	0.62	0.3	80.2
Fox River Q [1] (n = 101)		PCB17	16.95	3.87	0.84	0.18	89.1
Fox River Q [1] (n = 102)		PCB18+30	11.62	1.55	0.89	0.16	92.2
Fox River Q [1] (n = 91)		PCB105	9.15	2.05	0.58	0.59	70.3
Fox River Q [1] (n = 65)		PCB177	8.45	2.36	0.58	0.65	73.8
Fox River Q [1] (n = 48)		PCB183+185	6.33	1.81	0.61	0.64	68.8
Fox River Q [1] (n = 47)		PCB67	4.13	0.51	0.84	0.59	85.1
Fox River Q [1] (n = 62)		PCB11	-4.82	2.3	0.81	0.99	67.7
Fox River Q [1] (n = 33)		PCB128+162+166+167	- 10.48	2.61	0.92	0.54	100.0
Hudson River [3] (n = 84)	Time, flow, water temperature, season, site (random)	PCB6	0.92	0.32	0.69	0.6	77.4
Lake Michigan [4] (n = 94)	Time, water temperature, season, site (random)	PCB89	-1.74	0.54	0.39	0.41	77.7
Lake Michigan tributaries (n = 117)	Time, season, site (random)	PCB114+122+131+133+ 142+146+165	9.71	4.89	0.73	0.34	94.0
Lake Michigan tributaries (n = 154)		PCB4+10	-2.28	0.35	0.85	0.66	86.4
Lake Washington (n = 33)	Time, season, site (random)	PCB45+51	-0.96	0.44	0.8	0.22	87.9
New Bedford Harbor [5] (n = 42)	Time, site (random)	PCB129+137+138+158+ 160+163+164+176+178	7.81	2.85	0.15	0.5	42.9

New Bedford Harbor [5] (n = 50)		PCB132+153+161+168	5.96	1.14	0.49	0.59	56.0
New Bedford Harbor [5] (n = 41)		PCB180+193	5.02	1.43	0.57	0.64	58.5
New Bedford Harbor [5] (n = 81)		PCB43+49+52+69+73	4.56	0.52	0.81	0.41	74.1
New Bedford Harbor [5] (n = 72)		PCB20+21+28+31+33+ 50+53	4.46	0.48	0.76	0.6	65.3
New Bedford Harbor [5] (n = 41)		PCB182+187	4.27	1.13	0.55	0.68	51.2
New Bedford Harbor [5] (n = 75)		PCB5+8	4.05	0.44	0.76	0.61	57.3
New Bedford Harbor [5] (n =39)		PCB170+190	4.0	1.1	0.61	0.62	71.8
New Bedford Harbor [5] (n = 37)		PCB128+162+166+167	1.92	0.72	0.65	0.61	75.7
New Bedford Harbor [5] (n = 37)		PCB195+208	1.32	0.19	0.82	0.69	81.1
New Bedford Harbor [5] (n = 38)		PCB206	1.13	0.14	0.84	0.75	76.3
New Bedford Harbor [5] (n = 37)		PCB209	1.11	0.14	0.85	0.74	78.4
Passaic River (n = 101)		PCB155	5.83	2.67	0.82	0.51	100.0
Passaic River (n = 309)	Time, flow, water temperature, season, site (random)	PCB209	5.36	1.11	0.76	0.54	94.5
Passaic River (n = 132)		PCB189	-3.8	1.69	0.83	0.56	98.5
Passaic River Q (n = 132)		PCB189	-3.97	1.89	0.83	0.56	98.5
Passaic River Q (n = 159)		PCB3	- 16.09	5.92	0.54	0.39	91.2
Portland Harbor (n = 85)	Time, flow, water temperature, season, site (random)	PCB43+49+52+69+73	34.19	11.25	0.73	0.19	88.2
Portland Harbor (n = 85)		PCB44+47+65	25.67	8.36	0.67	0.26	75.3
Portland Harbor (n = 76)		PCB12+13	19.17	3.42	0.53	0.49	72.4
Portland Harbor (n = 85)		PCB132+153+161+168	16.1	3.9	0.67	0.31	65.9
Portland Harbor (n = 85)		PCB83+99	14.71	2.46	0.73	0.24	77.6
Portland Harbor (n = 85)		PCB88+91	13.67	2.1	0.81	0.22	81.2

Portland Harbor (n = 62)		PCB15	-0.06	0.02	0.36	0.34	79.0
Portland Harbor (n = 60)		PCB6	-0.07	0.04	0.32	0.37	65.0
Portland Harbor Q (n = 85)		PCB43+49+52+69+73	44.62	19.5	0.76	0.18	91.8
Portland Harbor Q (n = 85)		PCB44+47+65	28.62	10.81	0.68	0.26	74.1
Portland Harbor Q (n = 76)		PCB12+13	24.18	6.01	0.58	0.5	75.0
Portland Harbor Q (n = 85)		PCB132+153+161+168	17.64	4.83	0.67	0.3	70.6
Portland Harbor Q (n = 85)		PCB88+91	15.43	2.71	0.83	0.21	83.5
Portland Harbor Q (n = 62)		PCB15	-0.07	0.03	0.39	0.34	80.6
Portland Harbor Q (n = 59)		PCB67	-0.09	0.05	0.47	0.31	88.1
Spokane River Q (6) (n = 43)	Time, flow, season, site (random)	PCB209	-2.56	1.18	0.22	0.15	97.7

(1): Site Lake Winnebago removed from analysis due to be considered a background site and not located in Fox River.
(2): Site Plain Well Dam removed from analysis due to samples collected during sediment dredging.
(3): Sites upstream from known PCBs sources (Bakers Falls) were not included in the analysis.
(4): Only samples from Lake Michigan.
(5): Not enough samples from different season to include season as a covariate.
(6): Samples only collected from Spokane River.

References

Martinez, A. (2024). Dataset of surface water concentrations of Polychlorinated Biphenyls in the U.S. from 1979 - 2020 [dataset]. PANGAEA, https://doi.org/10.1594/PANGAEA.972705.

Martinez, A. (2024). R Code: PCB Water Analysis. Zenodo. https://doi.org/10.5281/zenodo.13887687.
